# Overview of Indian and Nepali representatives of the *Cincticostella nigra* (Uéno, 1928) complex (Ephemeroptera, Ephemerellidae), with discussion about *Cincticostella* Allen, 1971 species complexes

**DOI:** 10.3897/zookeys.1040.64280

**Published:** 2021-05-28

**Authors:** Alexander V. Martynov, C. Selvakumar, Dmitry M. Palatov, K.A. Subramanian, K.G. Sivaramakrishnan, M. Vasanth, Luke M. Jacobus

**Affiliations:** 1 National Museum of Natural History, National Academy of Sciences of Ukraine, Bohdan Khmelnytsky str., 15, 01030, Kyiv, Ukraine National Museum of Natural History, National Academy of Sciences of Ukraine Kyiv Ukraine; 2 Department of Zoology, The Madura College (Autonomous), Madurai-625011, Tamil Nadu, India Department of Zoology, The Madura College Madurai India; 3 A.N. Severtsov Institute of Ecology and Evolution of RAS, 33, Leninsky prospect 33, 119071, Moscow, Russia A.N. Severtsov Institute of Ecology and Evolution of RAS Moscow Russia; 4 Zoological Survey of India, Southern Regional Centre, Santhome High Road, Chennai-600028, India Zoological Survey of India, Southern Regional Centre Chennai India; 5 Flat 3, Gokulam Apartments, No.7, Gokulam Colony, West Mambalam, Chennai-600 033, India Unaffiliated Chennai India; 6 Division of Science, Indiana University Purdue University Columbus, Columbus, Indiana, 47203, USA Indiana University Purdue University Columbus Columbus United States of America

**Keywords:** China, Ephemerellinae, Ephemerelloidea, Indomalayan Region, new records, new species, Pannota

## Abstract

The concept of the *Cincticostella nigra* (Uéno, 1928) (Ephemeroptera: Ephemerellidae) complex is clarified and Indian and Nepali representatives of the complex are reviewed. Four new species are described viz. *Cincticostella changfai* Martynov & Palatov, **sp. nov.**, *Cincticostella funki* Martynov, Selvakumar, Palatov & Vasanth, **sp. nov.**, *Cincticostella shinichii* Martynov & Palatov, **sp. nov.** and *Cincticostella wangi* Selvakumar, Martynov & Subramanian, **sp. nov.** The larva of *C. corpulenta* (Braasch, 1981) is re-described, based on the holotype and paratypes. *Cincticostella gosei* (Allen, 1975) is recorded from India for the first time. Morphological differences of the species complexes of *Cincticostella* Allen, 1971 are analysed. A new species complex, *Cincticostella gosei* complex, is proposed. Summaries of distribution data and habitat preferences, as well as new larval diagnoses, are presented for all species of the *Cincticostella nigra* complex.

## Introduction

This article is a further contribution to a series of papers (Martynov et al. 2019, 2021) about spiny crawler mayflies (Ephemeroptera: Ephemerellidae) of the Indian subregion, in this case devoted to Indian and Nepali representatives of the genus *Cincticostella* Allen, 1971 (Ephemerellinae: Ephemerellini) that were not previously covered as part of the *C. insolta* (Allen, 1971) complex.

Due to uncertain relationships of species in the genus *Cincticostella* and possible polyphyly, we use the term “complex” to indicate distinctly different groups of species – the *C. insolta* complex (see Xie et al. 2009; Martynov et al. 2019; Zheng and Zhou 2021) and what we clarify here as the *C. nigra* (Uéno, 1928) complex, which corresponds to the group of nine species described by Xie et al. (2009) as having “the basic body pattern” for the genus. Up to now, the *C. nigra* complex in this sense has been comprised of the following nine species (Jacobus and McCafferty 2008; Xie et al. 2009): *C. colossa* Kang & Yang, 1995, *C. corpulenta* (Braasch, 1981), *C. elongatula* (McLachlan, 1875), *C. fusca* Kang & Yang, 1995, *C. gosei* (Allen, 1975), *C. levanidovae* (Tshernova, 1952), *C. nigra* (Uéno, 1928), *C. orientalis* (Tshernova, 1952) and *C. szechuanensis* Xie, Jia, Chen, Jacobus & Zhou, 2009. Amongst these, Xie et al. (2009) noted some differences from “the basic body pattern” of the larva, in that *C. levanidovae* and *C. orientalis* have reduced maxillary palps and *C. gosei* lacks maxillary palps. Zhang et al. (2020) subsequently described the male adult of *C. fusca* and remarked about its “novel ephemerellid form and possibly…new evolutionary trend in the group”, but they did not recognise a new species group or complex for it.

The *C. nigra* complex, as well as the family Ephemerellidae in general, has been relatively poorly investigated in India and Nepal. Only one species of the *C. nigra* complex has been reported from these countries: *C. corpulenta* described from Nepal by Braasch (1981), based on the larval stage. Another species, *C. indica* (Kapur & Kripalani, 1961) is known only from India, based on the original material of the female adult stage. Given that few adults of this genus are known and that female alates generally cannot be identified with confidence, we do not assign *C. indica* to a species complex; however, we treat it below.

## Material and methods

Materials from India and Nepal were used in this research. New larval material was collected by kick-net sampling and hand-picking. All of this material is stored in 80–95% ethanol. Some specimens were mounted on slides with Canada balsam or Hoyer’s medium.

The holotype of *C. corpulenta* from the Stuttgart State Museum of Natural History [**SSMNH**] (Stuttgart, Germany) was also examined, along with two paratypes from the Purdue University Entomological Research Collection [**PERC**] (West Lafayette, Indiana, USA). The type material of *C. changfai* sp. nov. (holotype and 31 paratypes – *IN Nepa5Cinsp1/1*; *Nepa5Cinsp1/2–4*, slide 640; *Indi2Cinsp*, slides 630, 657, 658), *C. shinichii* sp. nov. (holotype and 1 paratype – *IN Nepa7Cinsp*, slides 647, 649) and specimens of *Cincticostella* sp. A (two larvae – *IN Nepa1Cinsp*, slide 634) are now housed in the National Museum of Natural History of the National Academy of Sciences of Ukraine [**NMNHNASU**], Kyiv, Ukraine. Type material of *C. funki* sp. nov. is deposited in the Zoological Survey of India [**ZSI**], Chennai, India (holotype and one paratype – *IN ZSI/SRC-I/E-512*, *ZSI/SRC-I/E-513*) and in NMNHNASU (one paratype – *IN Indi1Cinsp*, slide 632); *C. wangi* sp. nov. (holotype and eight paratypes – *IN 5575/H13*) and *C. gosei* (17 larvae) are deposited deposited in ZSI, Kolkata.

Photographs of specimens and their body parts were taken using a Leica M205A microscope, Zeiss Stemi 2000 binocular with Canon Power Shot A 640 and Ulab XY-B2T microscope with Canon Power Shot A 630. Some figures were subsequently improved with Adobe Photoshop CS5 and Helicon Focus 6.

Our hypotheses of species are based on morphological species concepts. Two middle instar larvae from a single morphotype were collected in Nepal in 2014. These larvae belong to the *C. nigra* complex and slightly differ from all known species. However, the poor material and absence of late larval instars do not allow us to describe a new species adequately and with confidence. Therefore, we give a provisional species designation only, along with diagnostic features.

## Results and discussion

The analysis of original material, historical collections and literature data showed that there are seven species belonging to *C. nigra* and *C. gosei* complexes (see details below) within India and Nepal: *C. corpulenta* (Braasch, 1981), *C. gosei* (Allen, 1975), four new species (*C. changfai* Martynov & Palatov, sp. nov., *C. shinichii* Martynov & Palatov, sp. nov., *C. wangi* Selvakumar, Martynov & Subramanian, sp. nov. and *C. funki* Martynov, Selvakumar, Palatov & Vasanth, sp. nov.) and one provisional species that we designate as *Cincticostella* sp. A. The taxonomic status of *C. indica* (Kapur & Kripalani, 1961) is discussed below. In addition, we report the first record of *C. gosei* for India.

Diagnoses of species provide characters that distinguish them within their corresponding complexes.

The following nominal species are treated in alphabetical order, with the provisional species listed at the end.

### Cincticostella changfai

Taxon classificationAnimaliaEphemeropteraEphemerellidae

Martynov & Palatov sp. nov.

85139D40-F105-5A88-B3B3-02AD635F6A09

http://zoobank.org/C04D239A-E4B2-4E72-B238-3F42C3616861

Figs 1 , 2 , 3 , 4

#### Description.

**Figure 1.**
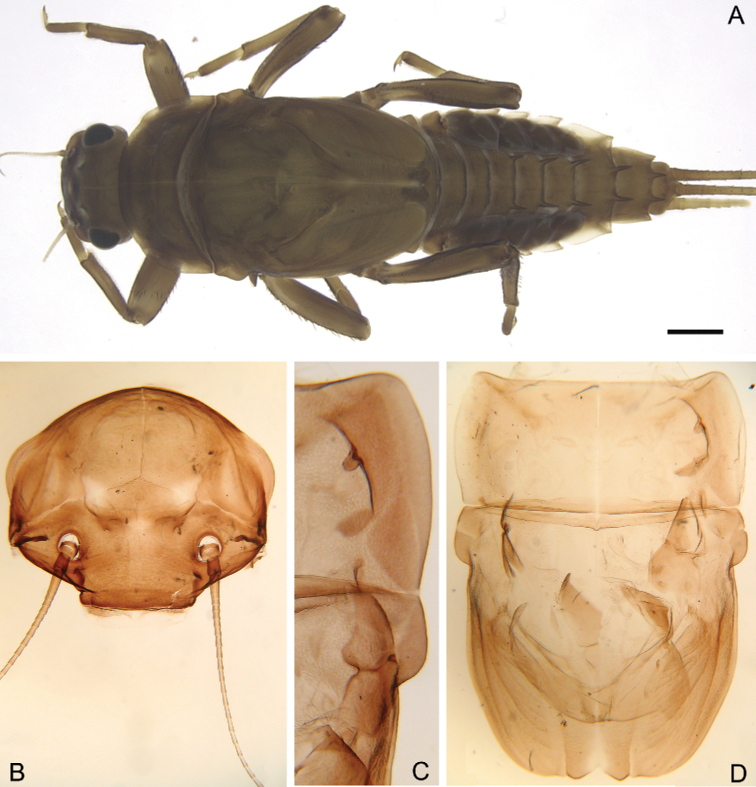
Larva of *Cincticostella changfai* Martynov & Palatov, sp. nov., paratype **A** habitus, dorsal view, scale bar 1 mm **B** head **C** anterolateral projections of pro- and mesonotum **D** thorax.

**Larva.** Late instars: body length 10.7–14.0 mm; caudal filaments length 6.0–9.1 mm. Body robust, yellowish-brown to brown (Fig. 1A).

**Figure 2.**
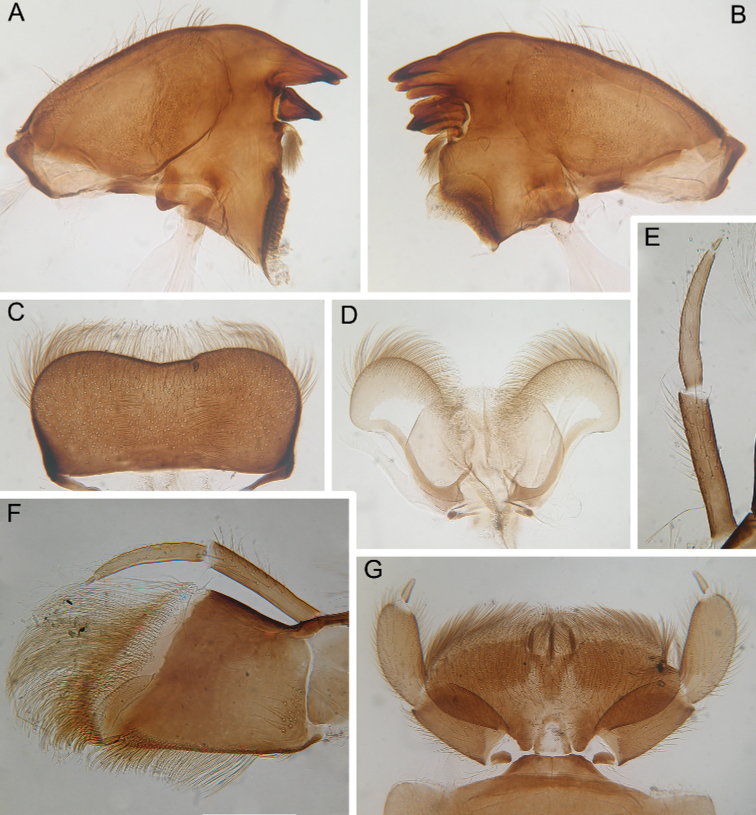
Larva of *Cincticostella changfai* Martynov & Palatov, sp. nov., paratype **A** planate mandible **B** angulate mandible **C** labrum **D** hypopharynx **E** maxillary palp **F** maxilla **G** labium.

***Head*:** Without tubercles and ridges. Genae moderately developed, rounded (Fig. 1B). *Mouthparts* (Fig. 2A–G): Labrum (Fig. 2C) wide, angles rounded; anteromedian emargination wide and shallow (labrum height in emargination/maximum labrum height ratio – 0.84–0.87); surface densely covered with long, stout hair-like setae, very short rounded scales with feathered margin and empty scale sockets; anterior margin and angles with thin and stout hair-like setae; central part of anterior margin also with feathered setae. Mandibles (Fig. 2A, B) with empty scale sockets, very short scales and long hair-like setae, the greatest number of the hair-like setae near outer margin; prostheca consisting of protuberance with tuft of middle-sized hair-like setae. Right (planate) mandible with row of long thick hair-like setae (5–8) under mola and tuft of short or middle-sized hair-like setae above; outer and inner incisors (kinetodontium) with three teeth. Left (angulate) mandible with few short hair-like setae at end of mola; outer incisor apex with four teeth; inner incisor with four teeth, the two central ones distinctly larger. Rounded apices of superlinguae with long stout and thin hair-like setae; surfaces of lingua covered with thin hair-like setae (Fig. 2D). Irregular rows of short, pointed, stout setae (up to 9) present on lingua surface near base; these rows mainly subtransverse relative to longitudinal axis of body. Maxilla (Fig. 2F) with two dentisetae, their inner margins serrate. Apex and apical part of maxilla surfaces with numerous long, stout hair-like setae; inner margin of galea-lacinia covered with dense row of stout hair-like setae; surface of galea-lacinia near base with group of 13–22 different-sized stout hair-like setae. Maxillary palp long, 3-segmented, with distinct articulations (Fig. 2E, F). Segments I and II with long, hair-like setae; most stout setae located on segment I; segment III short and rounded apically, with several fine setae. Labium with rounded glossae. Whole ventral surface of labium, dorsal surface of glossae and apices of paraglossae densely covered with long, stout, hair-like setae (Fig. 2G). Labial palp 3-segmented; segment I and segment II flattened and subequal in length, ventral side covered with long stout and thin hair-like setae; central part of segment I dorsal surface densely covered with empty scale sockets and very short rounded scales with feathered margins. Segment III elongated (length/width ratio = 2.66–3.5, average – 2.9), rounded apically, covered with numerous short fine setae.

***Thorax*:** Dorsal surface of thorax covered with few scattered short, hair-like setae. Pronotum expanded laterally, with broad, rounded, anterolateral projections (Fig. 1D). Anterolateral projections of mesothorax well-developed, not notched, broad, somewhat rounded, with outer margins not subparallel to lateral aspect of body (Fig. 1C). Thoracic surface without distinct ridges and tubercles. In mature larvae, paired posterior projections between fore wing pads moderately developed, triangulate, cleft between them wide; apical parts of outer margins of projections not pressed against wing pads.

**Figure 3.**
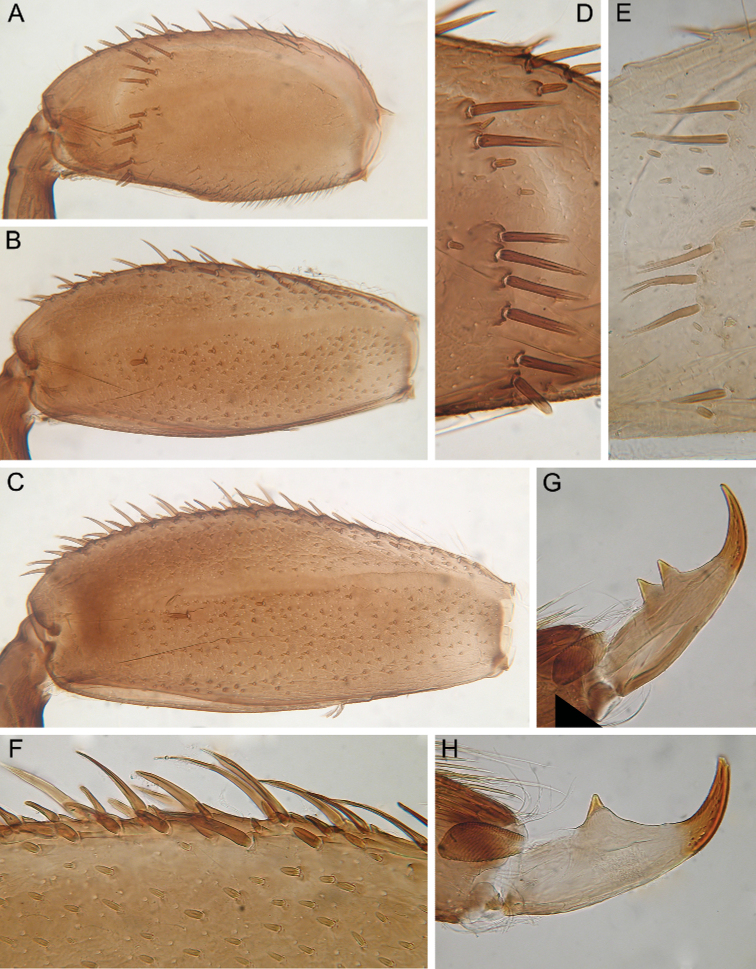
Larva of *Cincticostella changfai* Martynov & Palatov, sp. nov., paratype. **A** fore femur **B** middle femur **C** hind femur **D, E** stout setae of dorsal surface of fore femur **F** stout setae of outer margin of hind femur **G, H** tarsal claws.

In late instars, femora of all legs slightly flattened (length/width ratio = fore femur 2.17–2.38; middle femur 2.56–2.86; hind femur 2.63–2.86), each one with longitudinal ridge, especially visible on middle and hind femora (Fig. 3A–C). Average length ratio of leg’s parts (femur : tibia : tarsus): foreleg 1.90 : 1.63 : 1.00; middle leg 2.40 : 2.27 : 1.00; hind leg 2.59 : 2.77 : 1.00. Outer margins of all femora without apical projection.

Whole dorsal surface of fore femur with scattered middle-sized, hair-like setae; basal part of surface with group of spine-like setae, bifurcated stout setae and stout hair-like setae. Distal part of dorsal surface with narrow, transverse band consisting of irregular, sparse row of mainly long and middle-sized, pointed, bifurcated and only sometimes rounded apically, stout setae (some of them situated on chalazae) (Fig. 3A, D, E); also several short, rounded and bluntly pointed apically, stout setae situated in same band. Outer margin of fore femur and dorsal surfaces along it covered by different-sized stout setae with pointed, rounded or bifurcated apices and few hair-like setae; most hair-like setae in basal part. Inner margin of fore femur and adjacent part of dorsal surface densely covered with spine-like setae and stout hair-like setae; dorsal surface along inner margin with similar setae, but more scattered. Inner margins of fore tibia and tarsus and dorsal surfaces along those margins densely covered with spine-like setae and stout, hair-like setae. Dorsal surface of fore tibia also with row of long, pointed and bifurcated, stout setae and scattered hair-like setae. Outer margin of fore tibia and tarsus with few hair-like setae only (solitary and in tufts).

Dorsal surfaces of middle and hind femora covered with few hair-like setae and numerous short and several middle-sized rounded or bluntly pointed (sometimes bifurcated) apically stout setae (Fig. 3B, C, F). Inner margins of middle and hind femora without rows of stout setae, with several hair-like setae. Outer margins of middle and hind femora with irregular rows of different-sized (mainly long), pointed and bifurcated, stout setae (Fig. 3F); also with few solitary and in tufts hair-like setae; longest hair-like setae in basal parts of margins.

Inner margins of middle and hind tibiae and tarsi densely covered with spine-like setae and stout, hair-like setae; inner margins of middle and hind tibiae also with long, pointed and bifurcated, stout setae. Outer margins of middle and hind tibiae with rows of long, pointed and bifurcated, stout setae. Outer margins of middle and hind tarsi with hair-like setae (solitary and in tufts) only.

Ventral surfaces of all tibiae and tarsi with hair-like setae (solitary and in tufts) and long, thin, pointed, stout setae; stout setae situated in apical parts of tibiae and tarsi and along their inner margins.

Tarsal claws of all legs hooked, usually with two subequal denticles (seldom with one denticle) (Fig. 3G, H) and several subapical setae (setae often broken, as in our figures, but sockets still visible).

**Figure 4.**
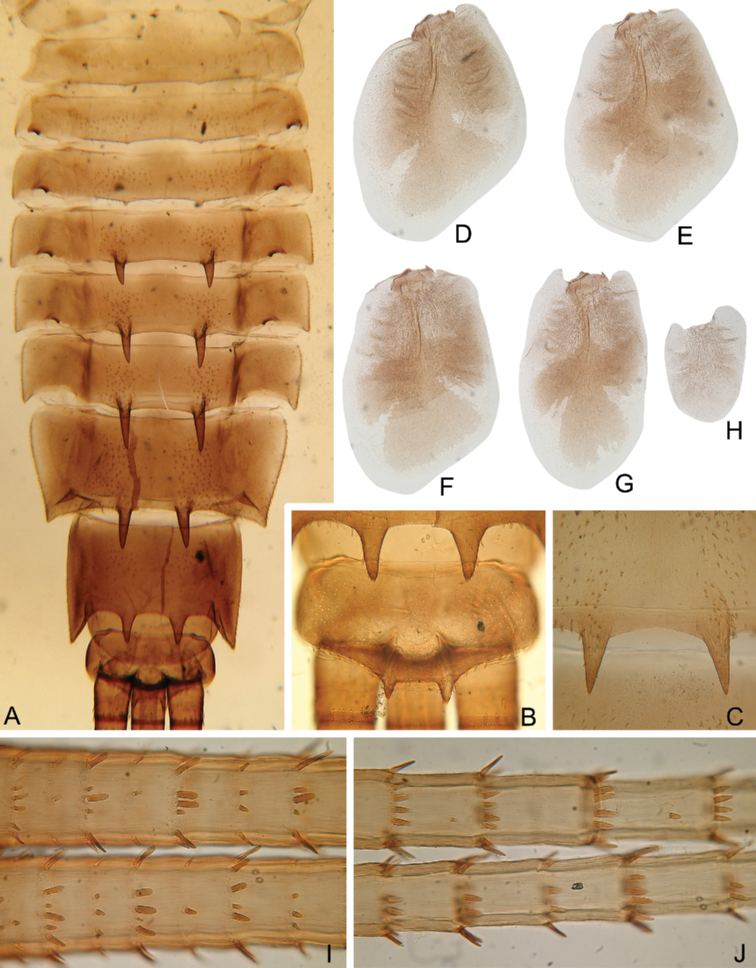
Larva of *Cincticostella changfai* Martynov & Palatov, sp. nov., paratype **A** abdomen, dorsal view **B** paired submedian projections of terga IX and X **C** paired submedian tubercles of tergum VIII **D–H** gills III–VII **I** caudal filaments, proximal part **J** caudal filaments, middle part.

***Abdomen*:** Central parts of dorsal surfaces of terga II–IX with two medial fields of middle-sized and small stout setae (Fig. 4A, C) with rounded, bluntly pointed or bifurcated apices. Surfaces of all terga covered with few hair-like setae, very short scales with feathery margins and empty scale sockets (most number of two last situated on lateral areas of tergal surfaces). Whole sternal surfaces covered with middle-sized hair-like setae, very short scales with feathery margins and empty scale sockets.

Posterior margins of terga I–II each with row of long hair-like setae; of tergum III with few long thin bluntly pointed or bifurcated apically stout setae and hair-like setae; of terga IV–VII with several rounded and pointed (sometimes bifurcated) stout setae near paired projections; of terga VIII–X with rows of short and middle-sized, mainly rounded apically, stout setae (these rows extending from paired projections to lateral margins). Lateral margins of terga IV–VIII covered with numerous short stout setae.

Pairs of pointed, not bifurcated projections present on abdominal terga II–X, with those on terga II–IV and X smaller than others; those on terga V–IX strongest (Fig. 4A–C). Projections on terga IX elongated and pointed. Surfaces of paired projections on terga V–X covered with short stout setae with mainly rounded apices. Posterolateral projections of abdominal segments IV–IX present.

Dorsal surfaces of abdominal gills covered with hair-like setae and scale sockets; shapes of gills as in Fig. 4D–H; gill III without medial, transverse band of weakened membrane; ventral lamella of gills III–V bifurcated; ventral lamella of gills VI without medial cleft.

Caudal filaments subequal in length, with elongated, bluntly pointed or rounded (sometimes bifurcated) apically stout setae and hair-like setae at articulations (Fig. 4I, J).

#### Adults.

Unknown.

#### Etymology.

The new species is named in honour of Dr. Chang-Fa Zhou (Nanjing Normal University, China), who contributed significantly to the study of the genus *Cincticostella*.

#### Diagnosis.

The new species can be easily distinguished from other representatives of the *C. nigra* complex by the following combination of characters: (i) genae moderately developed, rounded (Fig. 1B); (ii) labrum with shallow anteromedian emargination (Fig. 2C); (iii) maxillary palp well-developed (Fig. 2E, F); (iv) segment III of maxillary palp small, rounded apically (Fig. 2E); (v) prothoracic anterolateral projections small, broad, rounded (Fig. 1C, D); (vi) mesothoracic anterolateral projections well-developed, not notched, somewhat rounded, with outer margins not subparallel to lateral aspect of body (Fig. 1C, D); (vii) surface of thorax covered with a few scattered common, short, hair-like setae; (viii) fore femur with narrow, transverse band consisting of irregular, sparse row of mainly long and middle-sized, pointed, bifurcated and sometimes rounded apically, stout setae (Fig. 3A, D, E); also several short, rounded and bluntly pointed apically, stout setae situated in the band; (ix) tarsal claw with two subequal denticles mainly (Fig. 3G, H); (x) pairs of pointed, not bifurcated projections present on abdominal terga II–X, with those on terga II–IV and X smaller than others; those on terga V–IX strongest; projections on terga IX elongated and pointed (Fig. 4A–C).

#### Distribution.

Known only from Nepal and northern India.

#### Habitat.

Larvae of this species were collected in middle-sized rivers (wide 4–15 m) in deep valleys, at an altitude of about 1000 m a.s.l. on the South slope of the Great Himalaya Range (India, Uttarakhand State and Bagmati Zone, Central Nepal) (Fig. 24A). These rivers had low water temperature (12–13 °C), relatively high current velocity (about 0.7–0.8 m/s) and mainly stony bottom. Larvae were collected from stones at sections with current velocity 0.3–0.5 m/s together with *Baetis* (s.str.) sp., different Heptageniidae (*Electrogena*?), *Euthraulus* sp., Glossosomatidae and Hydropsychidae. The studied valleys of the rivers are densely populated by humans; therefore, the rivers are under the significant anthropogenic pressure. Investigated rivers can be classified as alpha- or beta-mezosaprobic waterbodies.

#### Type material.

***Holotype*: Nepal**: larva, Bagmati zone, Shivapuri Nagarjun National Park, Gohare Khola River (near Mahankal village), 27.885842°N, 85.531386°E, h ~ 1050 m a.s.l., 4.iii.2007, Chertoprud M.V. leg. – *IN Nepa5Cinsp1/1* [NMNHNASU]. ***Paratypes*: Nepal**: 22 larvae (one larva on slide 640), same data as holotype. – *IN Nepa5Cinsp1/2–4* [NMNHNASU]; **India**: 9 larvae (3 larvae on slides 630, 657, 658), Uttarakhand, Almora District, Ramganga River (300 m above Patangoari Village), 29.941569°N, 79.414394°E, h ~ 1050 m a.s.l., 3.ii.2011, Palatov D.M., Chertoprud M.V. leg. – *IN Indi2Cinsp* [NMNHNASU]. 6 larvae, Uttarakhand, Chamoli District, Pindar River (2 km above of the Karnaprayag Town), 30.251625, 79.229203, h ~ 780 m a.s.l., 4.ii.2011, Palatov D.M. leg. – *IN Indi4Cinsp1/1–3* [NMNHNASU].

### Cincticostella corpulenta

Taxon classificationAnimaliaEphemeropteraEphemerellidae

(Braasch, 1981)

70CE7CD2-C7F0-5185-8E7F-205E22B6D87A

Figs 5 , 6 , 7 , 8

Ephemerella (Drunella) corpulenta Braasch, 1981
Cincticostella corpulenta (Braasch, 1981) in Jacobus and McCafferty 2008

#### Remarks.

A supplemental narrative description is provided, based primarily on the study of a slide of the holotype (larva, male) and two paratypes in ethanol. Some characters are given simply as in the original description (Braasch 1981) due to subsequent damage, fading or distortion of the aging specimens.

#### Description.

**Figure 5.**
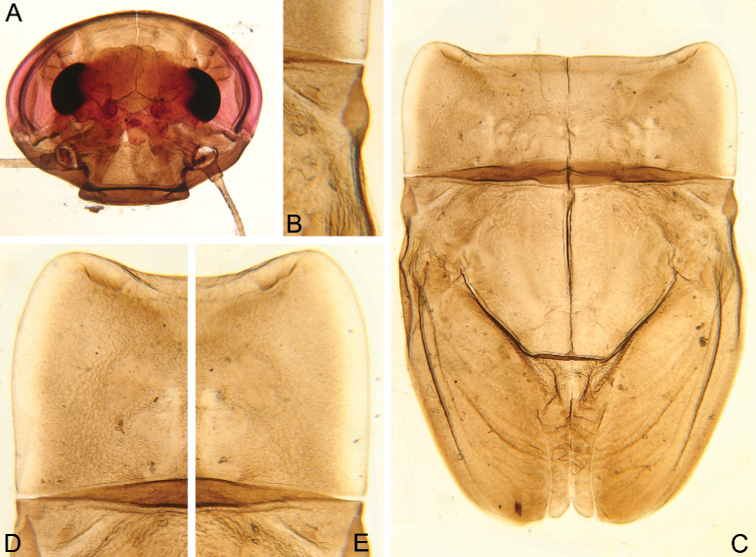
Larva of *Cincticostella corpulenta* (Braasch, 1981), holotype **A** head **B** anterolateral projection of mesonotum **C** thorax **D, E** anterolateral projections of pronotum.

**Larva (male).** Body brown according to Braasch (1981), body length 10 mm; caudal filaments length 6 mm. *Head*: Without tubercles and ridges. Braasch (1981) described head as elongated [original text in German: *Kopf langgestreckt*], but in holotype genae, these are poorly developed, therefore head appears oval dorsally (Fig. 5A).

**Figure 6.**
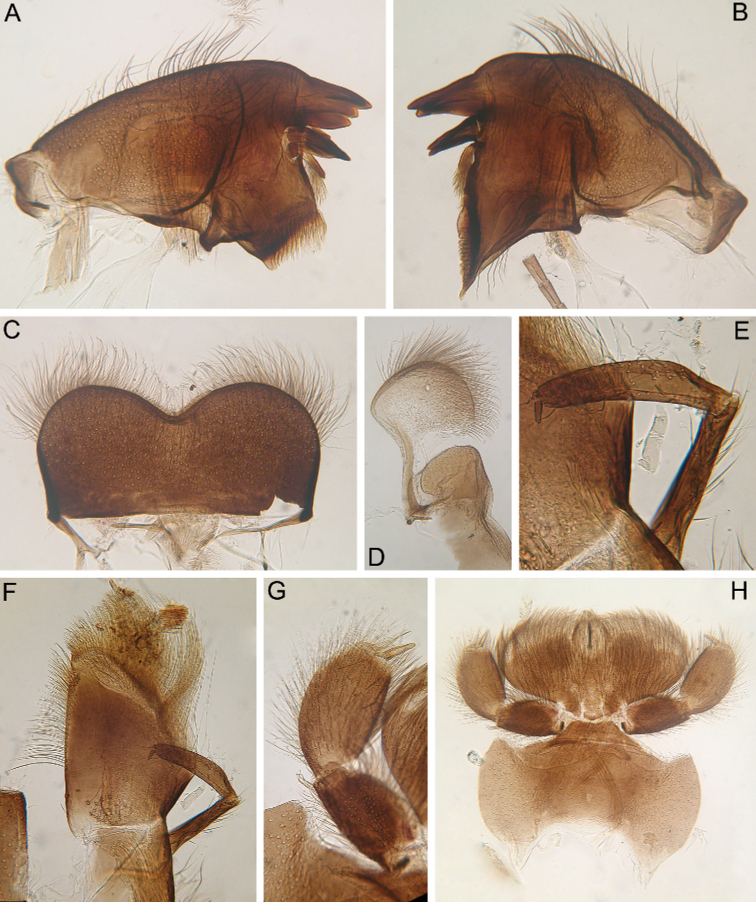
Larva of *Cincticostella corpulenta* (Braasch, 1981), holotype **A** angulate mandible **B** planate mandible **C** labrum **D** superlingua **E** maxillary palp **F** maxilla **G** labial palp **H** labium.

Dorsal surface of head covered with numerous very short scales, empty scale sockets and short, hair-like setae. *Mouthparts* (Fig. 6A–H): Labrum wide, angles rounded; anteromedian emargination deep (labrum height in emargination/maximum labrum height ratio – 0.73) (Fig. 6C). Anterior margin of labrum mainly with long, stout and thin, hair-like setae; dorsal surface densely covered mainly with long, stout hair-like setae (mainly in apical half), very short scales, empty scale sockets and short hair-like setae.

Mandibles (Fig. 6A, B) covered with empty scale sockets, very short scales and long hair-like setae (stout and thin), hair-like setae most numerous near outer margins and central parts of surfaces; both prosthecae consisting of protuberance with tuft of middle-sized hair-like setae. Planate mandible with row of six long, stout hair-like setae under mola and tuft of short hair-like setae above; outer incisor with three teeth; inner incisor (kinetodontium) bifurcated. Outer incisor of angulate mandible apex with four teeth; inner incisor with three teeth (two central ones distinctly larger). Superlinguae with rounded apices covered with long, stout hair-like setae (Fig. 6D); lingua with shorter setae. Lingua surface near base with transverse row of six short, pointed, stout setae. Maxilla (Fig. 6F) with two dentisetae with inner margins serrate. Apex and apical part of maxilla surfaces with numerous long, stout, hair-like setae; galea-lacinia with numerous, long, stout, hair-like setae on inner margin. Group (26, 29) of different-sized, stout, hair-like setae (some very long), situated on surface of galea-lacinia near base. Maxillary palp long, 3-segmented; joins between segments distinct (Fig. 6E). Segments I and II with long, hair-like setae, most strong setae situated on segment I; segment III slightly elongated and rounded apically. Glossae rounded; dorsal surfaces covered with short and middle-sized, stout, hair-like setae. Apices of paraglossae densely covered with mostly long and middle-sized, stout, hair-like setae; ventral surface of labium densely covered with mostly long, stout, hair-like setae (Fig. 6H). Labial palp 3-segmented (Fig. 6G); segment I and II distinctly flattened and subequal in length; ventral sides densely covered with long, hair-like setae (stout and thin); dorsal surfaces with long, stout, hair-like setae along outer and inner margins; central part of segment I dorsal surface also densely covered with scale sockets and very short rounded scales with feathered margins in some sockets. Segment III distinctly elongated (length/width ratio = 3.40–4.15), rounded apically, with short, fine setae mainly at apex. Submentum with scattered long, stout, hair-like setae, very short rounded scales with feathered margins in some sockets and empty scale sockets.

***Thorax*:** Dorsal surface of thorax covered with scattered short, waved and hooked, stout hair-like setae, thin and stout, hair-like setae and scale sockets (Fig. 23B); most distinct waved and hooked setae on veins of fore wing pads. Pronotum with moderately convex, rounded and broad anterolateral angles (Fig. 5C–E). Anterolateral projections of mesothorax poorly developed, rounded and not notched, with margins not subparallel to lateral aspect of body (Fig. 5B, C). Thoracic surface without distinct ridges and tubercles. Two blunt posterior projections present between fore wing pads; cleft between projections wide; apical parts of outer margins of projections pressed against wing pads.

**Figure 7.**
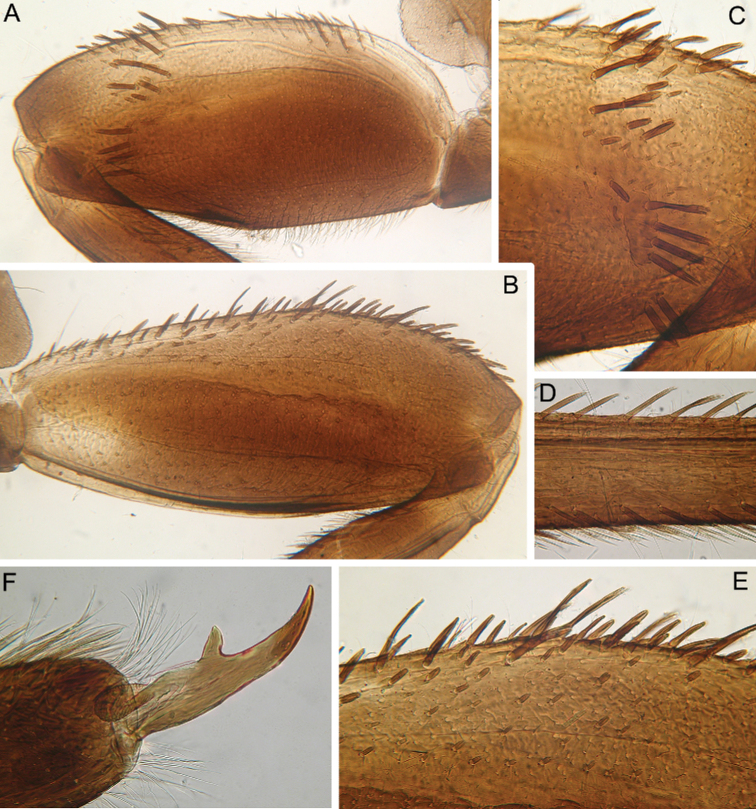
Larva of *Cincticostella corpulenta* (Braasch, 1981), holotype **A** fore femur **B** hind femur **C** stout setae of dorsal surface of fore femur **D** stout setae of hind tibia **E** stout setae of outer margin of hind femur **F** tarsal claw.

Legs slightly flattened (length/width ratio = fore femur 2.08; hind femur 2.56), each one with longitudinal ridge (Fig. 7A, B). Average length ratio of leg’s parts (femora : tibia : tarsi): foreleg 1.95 : 1.68 : 1; hind leg 2.59 : 2.38 : 1. Outer margins and proximal parts of dorsal surfaces of femora covered with irregular row of different-sized, stout setae with mostly rounded or bifurcated apices (Fig. 7A, B, E). Basal half of inner margin of fore femur and adjacent part of dorsal surface densely covered with spine-like setae and long, stout, hair-like setae. Apical half of dorsal surface of fore femur with transverse, sparse, band of mainly middle-sized and long, stout setae with bifurcated or rounded apices (some setae situated on chalazae) (Fig. 7A, C); several elongated, pointed or bifurcated apically, stout setae with feathered margins situated on dorsal surface near basal margin. Additionally, whole dorsal surface of fore femur covered with very short, rounded scales with feathered margins, empty scale sockets and different-sized hair-like setae. Inner margin and adjacent part of dorsal surface of fore tibia with numerous long, stout, hair-like setae and few, thin, hair-like setae (solitary and in tufts); another part of dorsal surface of fore tibia with row of long, bifurcated apically, stout setae and scattered hair-like setae (solitary and in tufts). Inner parts of ventral and dorsal surfaces of fore tibia and tarsus with numerous long, stout, hair-like setae (some with feathered margins in apical part); additionally, whole ventral and dorsal surfaces of fore tibia and tarsus with scattered hair-like setae (solitary and in tufts). Outer margins of fore tibia and tarsus with only long, hair-like setae. Outer margin of hind femur without apical projection; whole margin and part of dorsal surface covered with different-sized, stout setae with rounded and bifurcated apices. Whole dorsal surface covered with numerous short, rounded and bifurcated, stout setae, scale sockets, short scales with feathered margins in some sockets and scattered short, sometimes waved, stout, hair-like setae. Inner margin of hind femora with only solitary thin, hair-like setae. Outer margin of hind tibia with regular row of long, stout setae with pointed and bifurcated apices and scattered hair-like setae (Fig. 7B, E). Dorsal surface of hind tibia with irregular row of long, stout setae with bifurcated apices, situated along inner margin (Fig. 7D); whole surface covered with scattered long hair-like setae (solitary and in tufts) and short, stout, hair-like setae (some apparently waved or hooked). Inner margins and ventral surfaces of hind tibia and tarsus as in fore leg.

Tarsal claws of all legs hooked, with one large denticle and several subapical setae (Fig. 7F).

**Figure 8.**
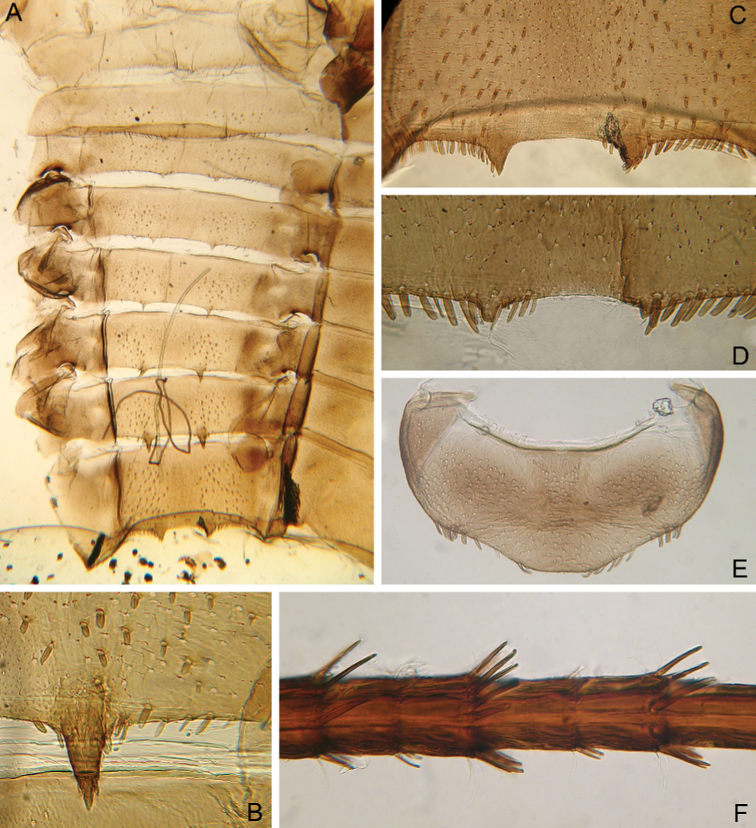
Larva of *Cincticostella corpulenta* (Braasch, 1981), holotype **A** abdomen on slide, dorsal view **B** submedian projection of tergum VII **C** paired submedian projections of tergum VIII **D** paired submedian projections of tergum IX **E** tergum X **F** caudal filament.

***Abdomen*:** Central part of dorsal surface of terga II–IX with two medial fields of mainly short stout setae with rounded or bluntly pointed, sometimes bifurcated, apices (Fig. 8A–C). Surfaces of all terga covered with not numerous hair-like setae (mainly stout, waved of hooked), empty scale sockets and very short rounded scales with feathered margins (most scales and sockets situated laterally). Whole sternal surfaces covered with scattered empty scale sockets, scales and hair-like setae. Posterior margins of tergum I with row of mostly long, hair-like setae; posterior margins of terga II–III (especially tergum II) with row of elongated, mostly bluntly pointed and bifurcated apically, stout setae and stout, hair-like setae; posterior margins of terga IV–VII with several elongated mostly rounded and bluntly pointed apically, stout setae, extending from paired projections to lateral margins; posterior margins of terga VIII–X with rows of elongated stout setae with mostly rounded apices (rows extending from paired projections to lateral margins) (Fig. 8B–E). Lateral margins of terga IV–VIII covered with rounded apically stout setae most numerous.

Pairs of not bifurcated projections present on abdominal terga II–IX, relatively weakly developed (Fig. 8A–E). Projections on terga VI–VIII strongest; those on terga II and III smallest and rounded; those on tergum IV elongated and bluntly pointed; those on terga V–VIII pointed; those on tergum IX short and bluntly pointed. Posterior margin of tergum X smooth, without any projections (Fig. 8E). Surfaces of paired projections on terga II–IX with several mostly short, stout setae with rounded apices.

Abdominal gills dorsal surfaces covered with hair-like setae and scale sockets; gill III oval, with somewhat extended posteromedial angles without medial, transverse band of weakened membrane. Due to slide-mounting of holotype, gills deformed and not separated from abdomen.

Caudal filaments subequal in length; middle parts with elongated stout setae with bluntly pointed or bifurcated apices (Fig. 8F) at articulations and hair-like setae (solitary and in tufts) on surfaces of segments.

#### Diagnosis.

This species can be distinguished from all other species of the *C. nigra* complex by the following combination of characters: (i) genae poorly developed, head oval dorsally (Fig. 5A); (ii) labrum with deep anteromedian emargination (Fig. 6C); (iii) maxillary palp well-developed (Fig. 6E); (iv) segment III of maxillary palp small, rounded apically (Fig. 6E); (v) prothoracic anterolateral projections rounded and broad (Fig. 5C–E); (vi) mesothoracic anterolateral projections poorly developed, rounded, not notched, with margins not subparallel to lateral aspect of body (Fig. 5B); (vii) short, waved and hooked, stout hair-like setae presented on dorsal surface of thorax (Fig. 23B); (viii) dorsal surface of fore femur with transverse, sparse, band of mainly middle-sized and long, stout setae with bifurcated or rounded apices (Fig. 7A and C); (ix) tarsal claw with one large denticle (Fig. 7F); (x) pairs of pointed, relatively weakly developed, projections present on abdominal terga II–IX; those on terga II and III smallest and rounded; those on terga VI–VIII strongest; those on terga V–VIII pointed; those on tergum IX short and bluntly pointed; posterior margin of tergum X smooth, without any projections (Fig. 8A–E).

#### Distribution.

Nepal (Braasch 1981).

#### Remarks.

Adult stages unknown. The holotype (on slide) has the middle legs and one hind leg missing.

#### Habitat.

No data, but assumed to be cold water rivers and streams, based on what we know about the Trisuli River near Dhunche.

#### Type material examined.

***Holotype*: Nepal**: larva on slide, Himalaya, Trisuli Khola vor Dhunche, 1950 m a.s.l. NN, 30.04.1978, Leg. I. Sivec [SSMNH]. ***Paratypes***: two larvae, same data as holotype [PERC].

### Cincticostella funki

Taxon classificationAnimaliaEphemeropteraEphemerellidae

Martynov, Selvakumar, Palatov & Vasanth sp. nov.

73452BC8-9EC3-5005-A09E-D8FCCA7ACF79

http://zoobank.org/D1E54A80-A4A5-421E-9612-C1E8C6077BA2

**Figure 9.**
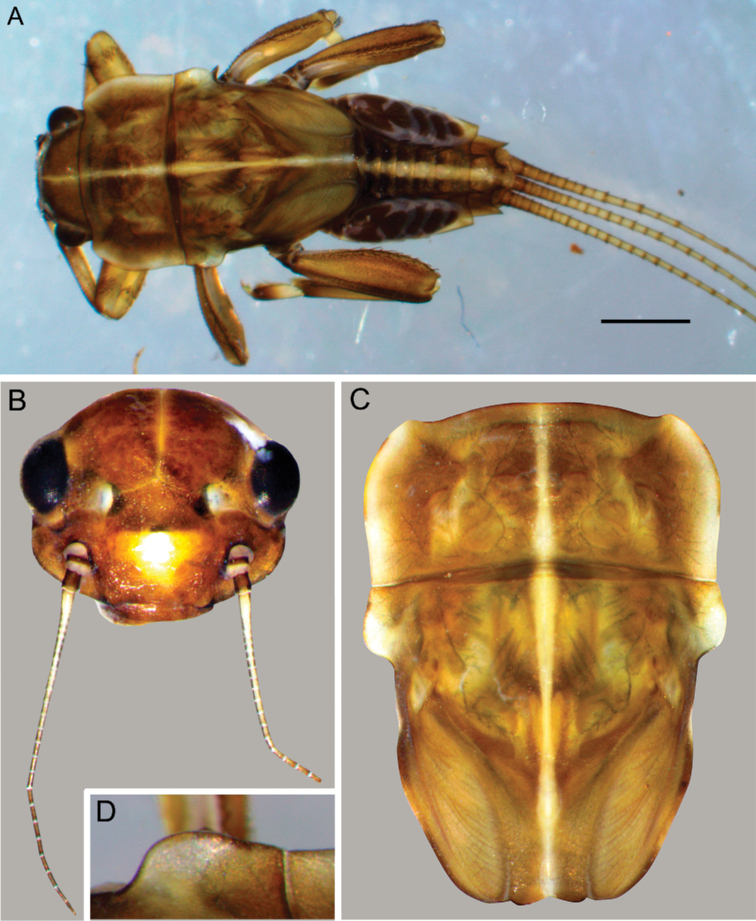
Larva of *Cincticostella funki* Martynov, Selvakumar, Palatov & Vasanth sp. nov., holotype (**A**) and paratype (**B–D**) **A** habitus, dorsal view, scale bar 2 mm **B** head **C** thorax **D** anterolateral projection of mesonotum.

**Figure 10.**
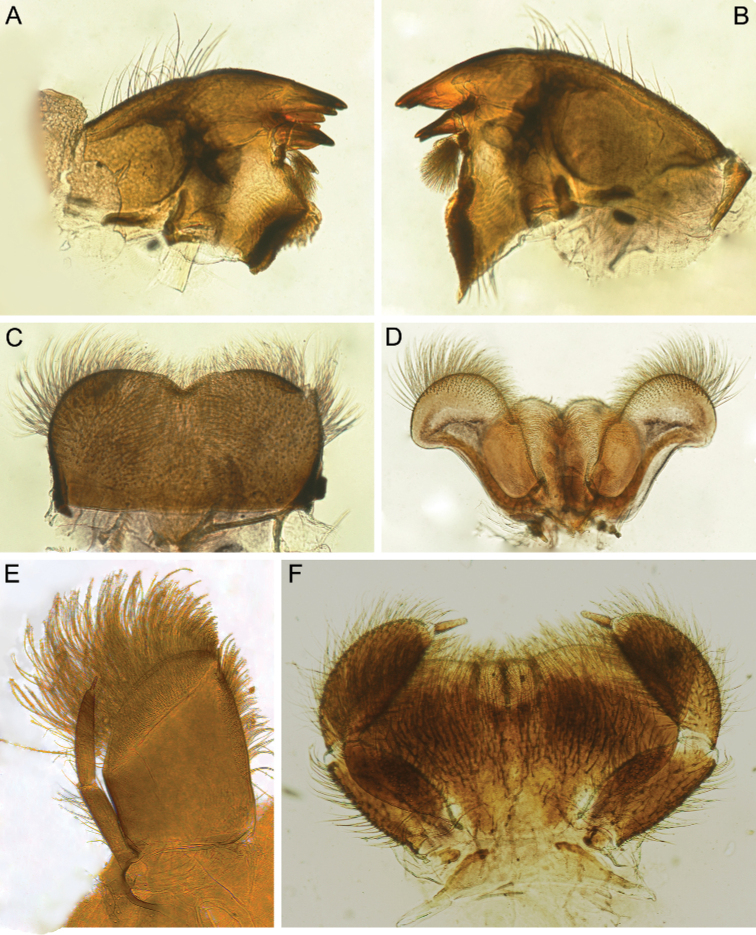
Larva of *Cincticostella funki* Martynov, Selvakumar, Palatov & Vasanth sp. nov., paratype **A** angulate mandible **B** planate mandible **C** labrum **D** hypopharynx **E** maxilla **F** labium.

**Figure 11.**
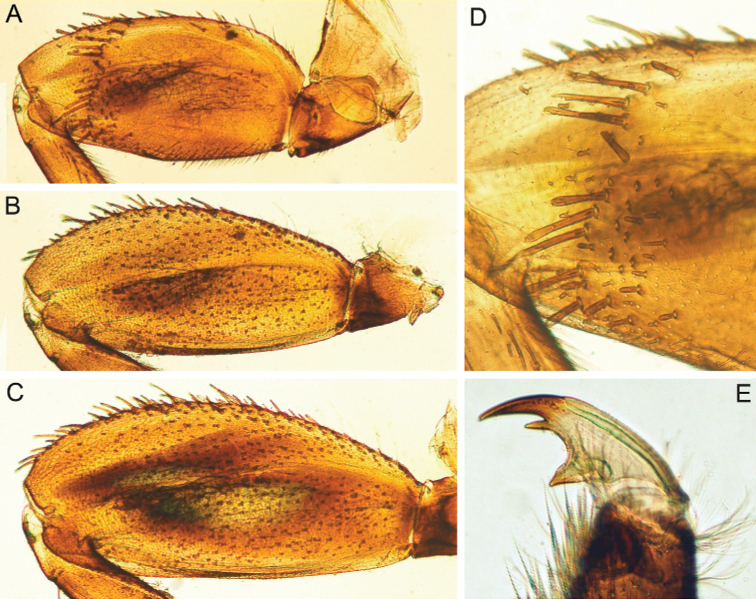
Larva of *Cincticostella funki* Martynov, Selvakumar, Palatov & Vasanth sp. nov., paratype **A** fore femur **B** middle femur **C** hind femur **D** stout setae of dorsal surface of fore femur **E** tarsal claw.

**Figure 12.**
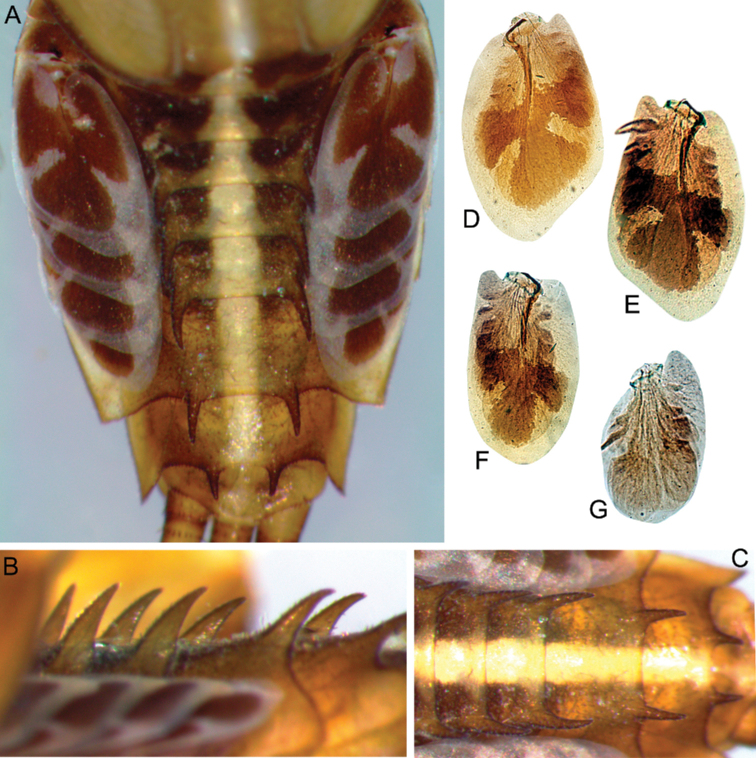
Larva of *Cincticostella funki* Martynov, Selvakumar, Palatov & Vasanth sp. nov., holotype (**A–C**) and paratype (**D–G**). **A** abdomen, dorsal view **B** paired submedian projections of terga VI–VIII, lateral view **C** paired submedian projections of terga V–IX, dorsal view **D–G** gills III–VI.

Figs 9 , 10 , 11 , 12

#### Description.

**Larva**. Late instars: body length 10.5–11 mm; caudal filaments length 6–7 mm. Body robust, yellowish-brown, abdomen darker (Fig. 9A). Head, thorax and abdomen with longitudinal medial white line; on abdomen line is most broad (Figs 9A–C, 12A, C). Anterolateral projections of mesothorax and lateral areas of pronotum are yellowish-whitish. Pronotum and mesonotum with indistinct whitish – yellowish spots.

***Head*:** Without tubercles and ridges. Genae rounded, moderately developed (Fig. 9B). *Mouthparts* (Fig. 10A–F): Anteromedian emargination of labrum shallow and narrow (Fig. 10C). Anterolateral angles of labrum rounded. Dorsal surface of the labrum covered mainly with long, thin and stout hair-like setae; setae decreasing in length towards central notch. In addition, setae with feathered margins, small hair-like setae and solitary empty scale sockets presented on labrum surface. Largest hair-like setae and their greatest number on mandibles situated in the centre of their outer margin and adjacent areas (Fig. 10A and B). Additionally, mandibles bearing few empty scale sockets. Prostheca consisting of protuberance with tuft of middle-sized, hair-like setae. Planate mandible with row of long thick hair-like setae (7–8) under mola and tuft of short or middle-sized hair-like setae above; outer and inner incisors (kinetodontium) with three teeth. Angulate mandible with few short hair-like setae at the end of mola; outer incisor apex with four teeth; inner incisor with four teeth, two central teeth distinctly larger. Apices of superlinguae rounded, bearing long, thin and stout, hair-like setae; surfaces of lingua covered with thin hair-like setae (Fig. 10D). Lingua with shallow medial concave on anterior margin. Surface of lingua with irregular, subtransverse to longitudinal axis of the body, rows of short, pointed, stout setae (8–9). Maxilla with two dentisetae; their inner margins serrated. Apical part of maxilla densely covered with long, stout hair-like setae (Fig. 10E). Inner margin of galea-lacilia with a dense row of stout hair-like setae, longest setae situated proximal. In addition, a group of 16–24 long, stout setae situated on surface of galea-lacilia base. Maxillary palp long, 3-segmented, with distinct articulation (Fig. 10E). Segment III elongated with several fine setae, bluntly pointed apically. Segments I and II subequal in a length, covered mainly with a long, stout hair-like setae. Labium robust, glossae rounded; labial palp 3-segmented (Fig. 10F). Segments I and II flattened, subequal in length; on dorsal surfaces of segments stout setae (long, hair-like) present mainly near margins; ventral surfaces, outer and inner margins of segments densely covered with long, stout, hair-like setae; central part of dorsal surface near inner margin on segment I with scale sockets with feathery setae. Segment III narrow, elongated (length/width ratio = 2.5–2.7), with rounded apex, covered with short fine setae. Whole ventral surface of labium covered mainly with long, stout, hair-like setae; dorsal surface of glossae and apices of paraglossae covered mainly with similar kind of setae.

***Thorax*:** Dorsal surface of thorax covered with scattered mainly short, waved and hooked, stout hair-like setae (Fig. 23B). Pronotum expended laterally; anterolateral projections broad, small and rounded (Fig. 9C). Anterolateral projections of mesothorax well-developed, with rounded posterior angles, outer margins not notched and not subparallel to lateral aspect of body (on slide with immature larva – subparallel) (Fig. 9C, D). Thoracic surface without any distinct ridges and tubercles. In mature larvae, paired posterior projections between fore wing pads small, rounded with narrow cleft between them; apical parts of outer margins of projections not pressed against wing pads (Fig. 9C).

All femora slightly flattened (length/width ratio = fore femur 2.0–2.1; middle femur 2.0–2.2; hind femur 2.0–2.2) (Fig. 11A–C), each one with longitudinal ridge, especially visible on middle and hind femora. Average length ratio of leg’s parts (femora: tibia : tarsi): foreleg 1.48: 1.43 : 1.00; middle leg 1.61 : 1.72 : 1.00; hind leg 2.10 : 2.32 : 1.00. All femora outer margins without apical projections, any distinct serration also absent.

Whole dorsal surface of fore femur covered with scattered middle-sized, hair-like setae and scale sockets with small scales in some of them; basal part of surface with group of spine-like setae, bifurcated, stout setae and stout, hair-like setae. Additionally, numerous long, bifurcated apically, stout setae covering dorsal surface of fore femora along basal half of its inner margin. Distal part of surface with relatively wide, transverse, band consisting of irregular, sparse rows of different-sized (mainly long), rounded apically (only sometimes bluntly pointed with shallow bifurcation of apex), stout setae; some of setae situated on chalazae (Fig. 11D). Outer margin of fore femur and dorsal surfaces along it covered with different-sized stout setae with long rounded and bifurcated apices and numerous middle-sized hair-like setae. Inner margins of fore tibia and tarsus and dorsal surfaces along those margins densely covered with spine-like setae and stout, hair-like setae. Dorsal surface of fore tibia also with row of long, pointed thick setae, stout setae and scattered hair-like setae. Outer margin of fore tibia and tarsus with few hair-like setae only (solitary and in tufts).

Dorsal surface of middle and hind femora covered with few scattered hair-like setae and scale sockets with small scales in some of them; also, surface covered with numerous short, rounded apically, stout setae (Fig. 11B, C). Inner margins of middle and hind femora without stout setae, only solitary hair-like setae present. Outer margins of middle and hind tibiae with moderately dense irregular rows of mainly long and pointed, stout setae and few hair-like setae amongst them.

Inner margins of middle and hind tibiae and tarsi densely covered with spine-like setae and stout, hair-like setae; inner margins of middle and hind tibiae also with long, pointed and stout setae. Outer margin of middle and hind tibiae with irregular row of long, pointed and bifurcated, stout setae. Outer margin of middle and hind tarsi with hair-like setae (solitary and in tufts) only.

Ventral surfaces of all tibiae and tarsi with hair-like setae (solitary and in tufts) and long, thin, pointed, stout setae; stout setae situated in apical parts of tibiae and tarsi and along their inner margins.

Tarsal claw of all legs hooked, with two (rarely three) denticles distanced from each other; basal denticle (rarely two denticles) distinctly larger; distal denticle directed angled forward (Fig. 11E); claw also bears several subapical setae.

***Abdomen*:** Posterior margins of tergum I with thin and stout, hair-like setae only; several similar setae present on surface of tergum I. Submedian areas of terga II–IX surfaces, posterior margins of terga VIII–X (excluding central area between submedian projections) and all paired submedian projections covered with small and middle-sized, oval or, sometimes, with slightly divergent margins, stout setae with rounded apices. Additionally, all terga surfaces covered with scattered, small, stout hair-like setae, short, thin, hair-like setae, scale sockets and short scales in some of them. Sterna covered with scattered thin and stout, hair-like setae and scale sockets.

Pairs of pointed, not bifurcated, projections present on abdominal terga II–IX; those on terga V–VIII strongest; those on terga II–IV distinctly smaller than others (Fig. 12A–C). Posterior margin of tergum X smooth, without projections. Abdominal segments IV–IX with posterolateral projections; on segment IX, they are most developed and directed backwards and laterally. Posterior margins of terga VIII–X with rows of short, stout setae with rounded apices (these setae almost absent in central areas of the margins). Lateral margins of terga IV–VIII covered with small stout setae with apices rounded.

Dorsal surface of abdominal gills covered with scattered hair-like setae and scale sockets; shape of gills as in Fig. 12D–G; medial, transverse band of weakened membrane absent on gill III.

Caudal filaments subequal in length, with mainly elongated, rounded apically, stout setae and hair-like setae at articulations.

#### Adults.

Unknown.

#### Etymology.

The new species is named in honour of Dr. David Funk (Stroud Water Research Center, USA), who contributed significantly to the study of Ephemerellidae.

#### Diagnosis.

The species is morphologically close to *C. shinichii* sp. nov. (see below), but can be distinguished from this species and other representatives of the complex by the following combination of the characters: (i) genae rounded and moderately developed (Fig. 9B); (ii) anteromedian emargination of labrum shallow and narrow (Fig. 10C); (iii) maxillary palp well-developed (Fig. 10E); (iv) segment III of maxillary palp elongated, rounded apically (Fig. 10E); (v) anterolateral projections of prothorax small, broad and rounded (Fig. 9C); (vi) mesothoracic anterolateral projections well-developed, with rounded posterior angles, outer margins not notched and not subparallel to lateral aspect of body (on slide with immature larva – subparallel) (Fig. 9C, D); (vii) surface of thorax covered with scattered mainly short, waved and hooked, stout hair-like setae (Fig. 23B); (viii) fore femur with relatively wide, transverse band consisting of irregular, sparse rows of different-sized (mainly long), rounded apically (only sometimes bluntly pointed with shallow bifurcation of apex), stout setae (Fig. 11A, D); (ix) tarsal claw with two denticles distanced from each other; basal denticle distinctly larger; distal one directed angled forward (Fig. 11E); (x) pairs of pointed projections present on abdominal terga II–IX; those on terga II–IV distinctly smaller than others; projections on tergum IX elongated and distinctly pointed; posterior margin of tergum X without paired projections (Fig. 12A–C).

#### Distribution.

Northern India and the India-China border region (Uttarakhand State and Arunachal Pradesh).

#### Habitat.

In Arunachal Pradesh, larvae of *C. funki* sp. nov. were collected from the Rike River (type locality) (2–5 m wide) at intermediate high mountain areas, on the Eastern Himalayan Range (Fig. 24B). Apparently, they prefer lotic water bodies with relatively low water temperature (9 °C–10 °C in sampling period), average current velocity (0.4–0.7 m/s), the bottom of river containing pebbles, cobbles and low level of silt particles and there is a median degree of anthropogenic pressure. Rivers inhabited by the species can be characterised as oligosaprobic. Larvae were collected from silted gravel and cobbles. Larvae of the mayfly *Baetis* sp., stoneflies Nemouridae and Peltoperlidae and caddisflies *Himalopsyche* sp. and *Rhyacophila* sp., were registered in the microhabitats along with *C. funki* sp. nov.

In Uttarakhand State, larvae of *C. funki* sp. nov. were collected from a small river (2–4 m wide) in medium high mountains, on the southern slope of the Great Himalayan Range (Fig. 24C). Apparently, they prefer small lotic waterbodies with relatively low water temperature (12 °C in sampling period), average current velocity (0.3–0.6 m/s), mosaic bottom and low degree of anthropogenic pressure. Rivers inhabited by the species can be characterised as oligosaprobic. Larvae were collected from silted gravel of the mayfly *Caenis* sp., stoneflies *Mesonemoura* sp. and *Kamimuria* sp. and the caddisfly *Lepidostoma* sp.

#### Type material.

***Holotype*: India**: larva, Arunachal Pradesh, Papumpare District, vicinity of Parang Village, Rike River, 27.32797°N, 93.50308°E, h ~1285 m a.s.l., 14.xii.2018, Coll. Bikramjit Sinha – IN *ZSI/SRC-I/E-512* [ZSI]. ***Paratypes*: India**: one larva, same data as holotype – *IN ZSI/SRC-I/E-513* [ZSI]. One larva (on slide 632), Uttarakhand, Almora District, 2^nd^ order left tributary of Ramganga River (in Dwarahat forest, 10.1 km north-eastwards of Chaukhutia Town), 29.925608 N, 79.445983 E, h ~ 1200 m a.s.l., 2.ii.2011, Palatov D.M. leg. – *IN Indi1Cinsp* [NMNHNASU].

### Cincticostella gosei

Taxon classificationAnimaliaEphemeropteraEphemerellidae

(Allen, 1975)

6B152F9E-1CC4-565E-A2B1-B9B1CF91E3EF

Figs 13 , 14 , 15

Ephemerella (Cincticostella) gosei Allen, 1975
Serratella thailandensis Allen, 1980 (junior objective synonym, Edmunds and Murvosh 1995)

#### Diagnosis.

**Figure 13.**
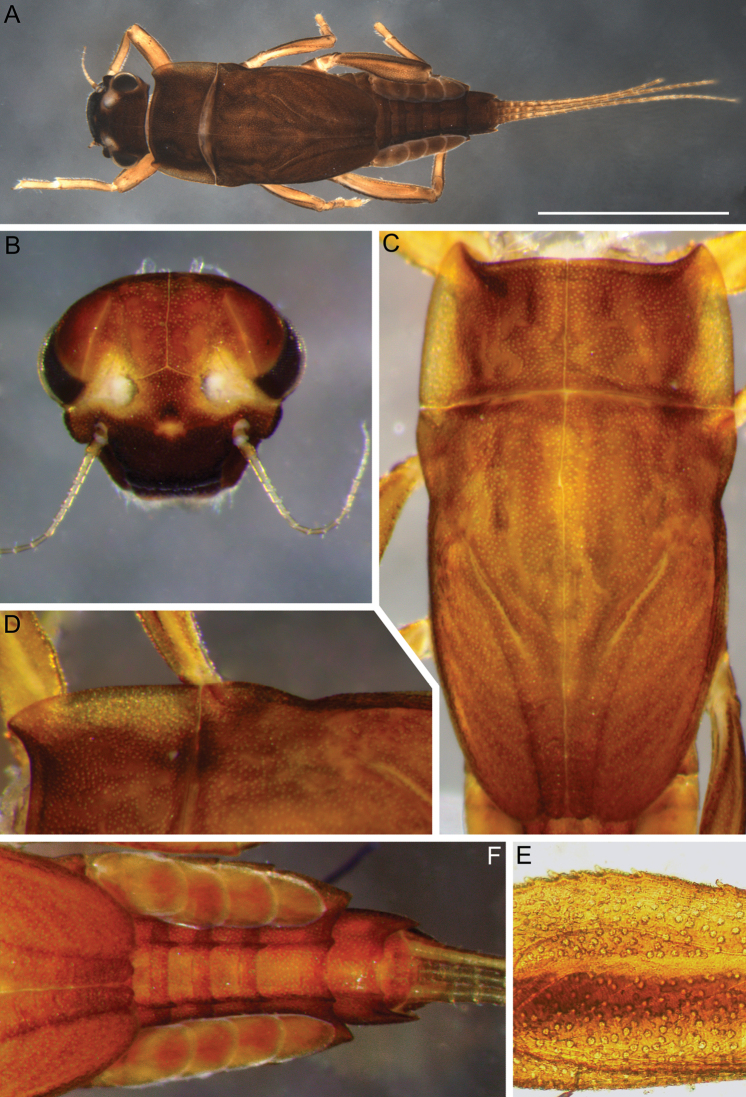
Larva of *Cincticostella gosei* (Allen, 1975) **A** habitus, dorsal view, scale bar 2 mm **B** head **C** thorax **D** anterolateral projections of pro- and mesonotum **E** dorsal surface of hind femur central area **F** abdomen, dorsal view.

**Figure 14.**
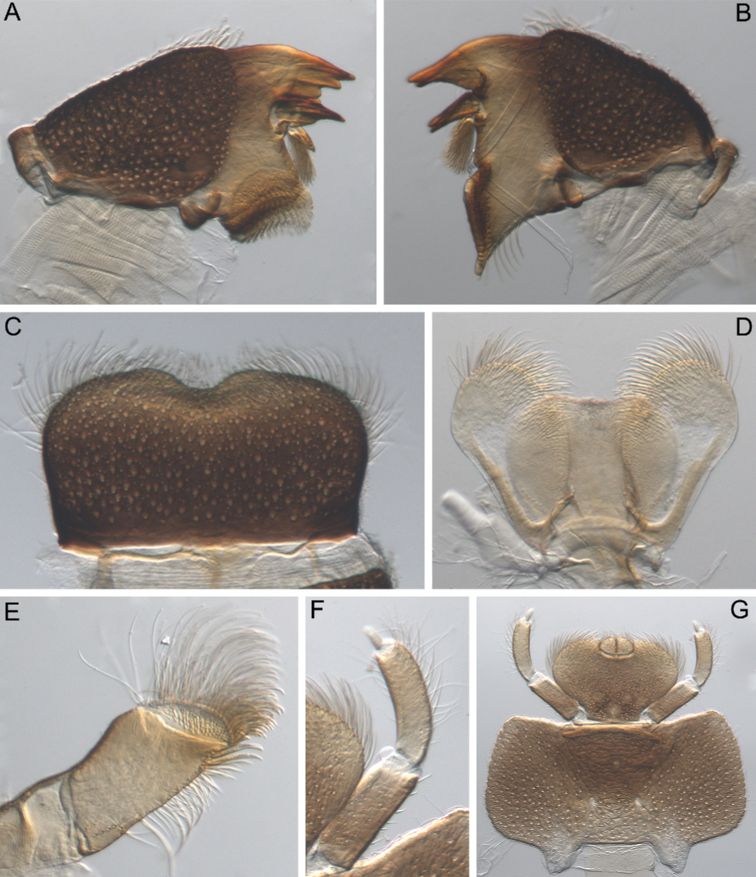
Larva of *Cincticostella gosei* (Allen, 1975) **A** angulate mandible **B** planate mandible **C** labrum **D** hypopharynx **E** maxilla **F** labial palp **G** labium.

**Figure 15.**
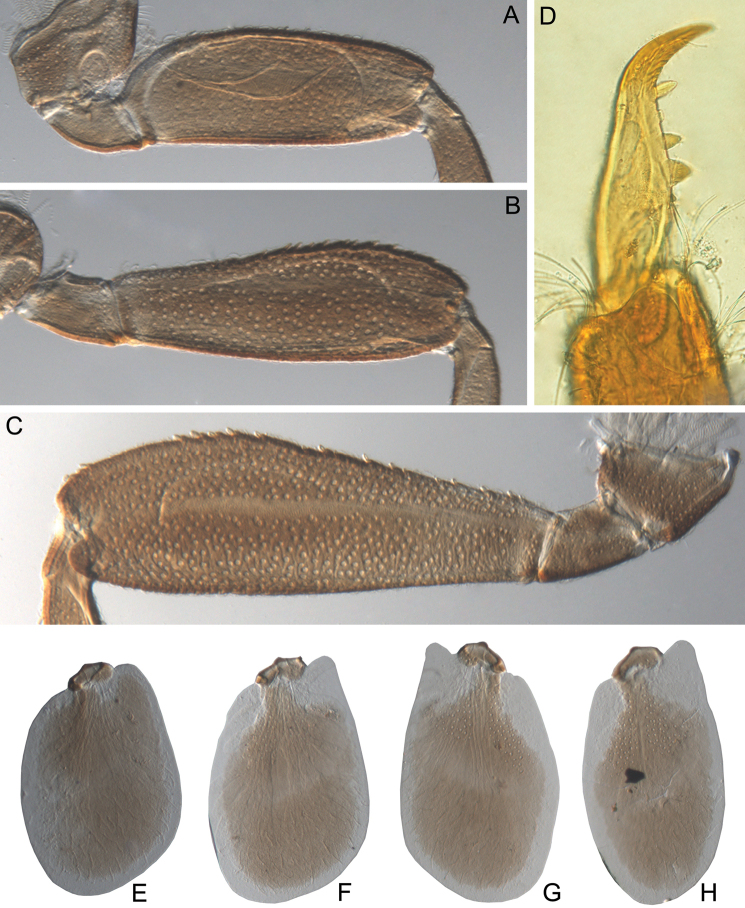
Larva of *Cincticostella gosei* (Allen, 1975) **A** fore femur **B** middle femur **C** hind femur **D** tarsal claw **E–H** gills III–VI.

This species can be distinguished from other *Cincticostella* species by the following combination of characters: (i) head brown, with three white to yellow spots near ocelli (spots near lateral ocelli the largest) (Fig. 13A, B); (ii) body covered with numerous large scale sockets and small scales in some of them (Figs 13B–E, 14A–C, 15A–C); (iii) head without paired protuberances (Fig. 13B); (iv) genae moderately developed (Fig. 13B); (v) labrum with moderate anteromedian emargination (Fig. 14C); (vi) maxillary palp absent (Fig. 14E); (vii) glossae short, inner margins of paraglossae subparallel to longitudinal axis of body, held tightly against glossae (Fig. 14G); (viii) labial palp segments I and II not flattened, elongated (Fig. 14F, G); (ix) prothoracic anterolateral projections well-developed, bluntly pointed, directed forward (Fig. 13C, D); (x) mesothoracic anterolateral projections poorly developed, not notched, rounded (Fig. 13C, D); (xi) setal transverse band on dorsal surface of fore femur consisting of only several middle-sized stout setae; sometimes stout setae absent absolutely; (xii) inner margin of fore femur only with scattered, thin, hair-like setae (Fig. 15A); (xiii) middle and hind femora moderately widened (Fig. 15B, C); (xiv) dorsal surfaces of middle and hind femora without stout setae (Figs 13E, 15B, C); (xv) outer margins of middle and hind femora without expressed projections (Fig. 15B, C); (xvi) tarsal claw with 5–6 denticles and several subapical setae (Fig. 15D); (xvii) small paired submedial projections present on terga II–IX; projections on tergum IX broad and rounded (Fig. 13F).

#### Distribution.

Thailand (Allen 1975, 1980) and the India-China border region (new data).

#### Remarks.

The larva of this species was properly described from Thailand by Allen (1975, 1980). We report this species for the first time from India. Adult stages are unknown. Main distinguishing characters of species are shown on Figs 13–15. The characters typical for representatives of *C. insolta* and *C. nigra* complexes are analysed because of the separate position of this species (see below).

#### Habitat.

Cold fast-flowing river with cobbles and gravel. The Ranga River habitat is shown in Martynov et al. (2019: fig. 152).

#### Material examined.

**India**: 17 larvae, Arunachal Pradesh, Lower Subansiri District, Ranga River, 27.396404°N, 93.757378°E, h ~ 625 m a.s.l., 06.xi.2015, Coll. Bikramjit Sinha – *IN 5346/H13* [ZSI].

### Cincticostella indica

Taxon classificationAnimaliaEphemeropteraEphemerellidae

(Kapur & Kripalani, 1961), nomen dubium, new status

A6CE9F0B-5080-56CF-928A-20F456012DC8

Ephemerella indica Kapur & Kripalani, 1961
Cincticostella indica (Kapur & Kripalani, 1961) in Jacobus and McCafferty 2008

#### Remarks.

*Cincticostella indica* was described only from the female adult stage (Kapur and Kripalani 1961) and it was included in *Cincticostella* by Jacobus and McCafferty (2008), based on the colouration of the abdomen of the female adult, which is similar to that of several other *Cincticostella* species. Its status was regarded as questionable by Xie et al. (2009). Recently, we tried to examine the holotype of *C. indica*, but the type specimen could not be located from the Zoological Survey of India, Kolkata (Hubbard and Srivastava 1984). Hence, *C. indica* cannot be identified and, given that the type material is apparently lost, we hereby regard it as a *nomen dubium*.

### Cincticostella shinichii

Taxon classificationAnimaliaEphemeropteraEphemerellidae

Martynov & Palatov sp. nov.

4FBF331B-AE4A-5FE1-8CF9-0F351B76E378

http://zoobank.org/04CA8710-E234-49FC-8822-AE53DEFD6F08

**Figure 16.**
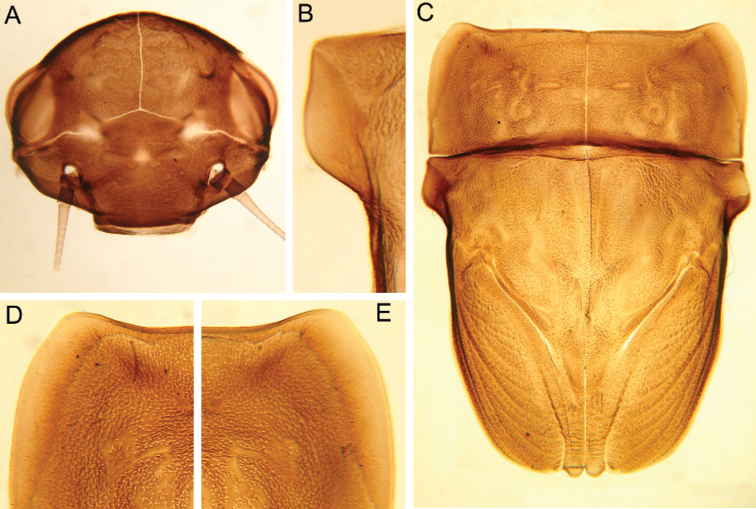
Larva of *Cincticostella shinichii* Martynov & Palatov, sp. nov., holotype (**A, B, D, E**) and paratype (**C**) **A** head **B** anterolateral projection of mesonotum **C** thorax **D, E** anterolateral projections of pronotum.

**Figure 17.**
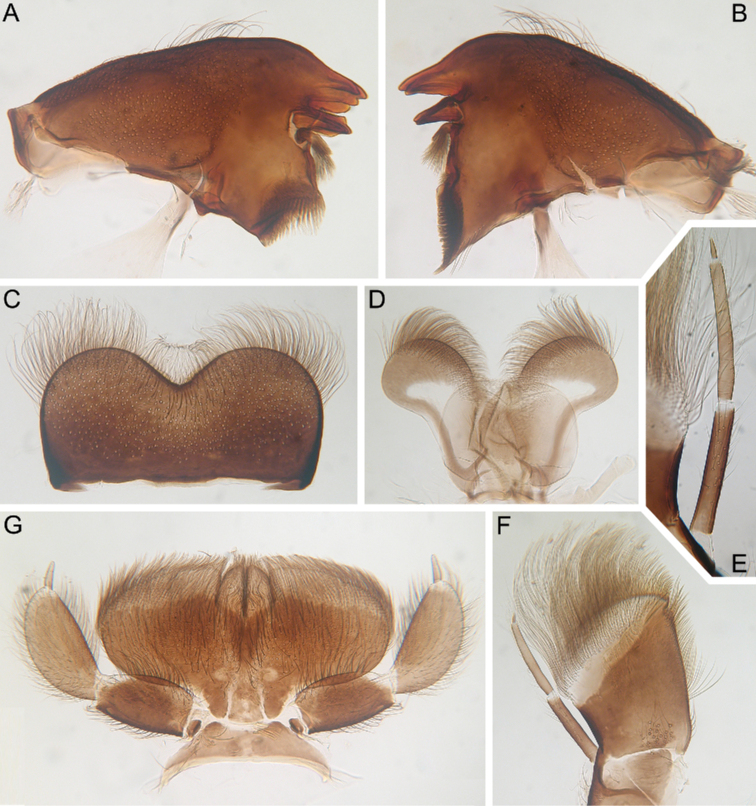
Larva of *Cincticostella shinichii* Martynov & Palatov, sp. nov., paratype (**A, B, G**) and holotype (**C–F**) **A** angulate mandible **B** planate mandible **C** labrum **D** hypopharynx **E** maxillary palp **F** apical half of maxilla **G** labium.

**Figure 18.**
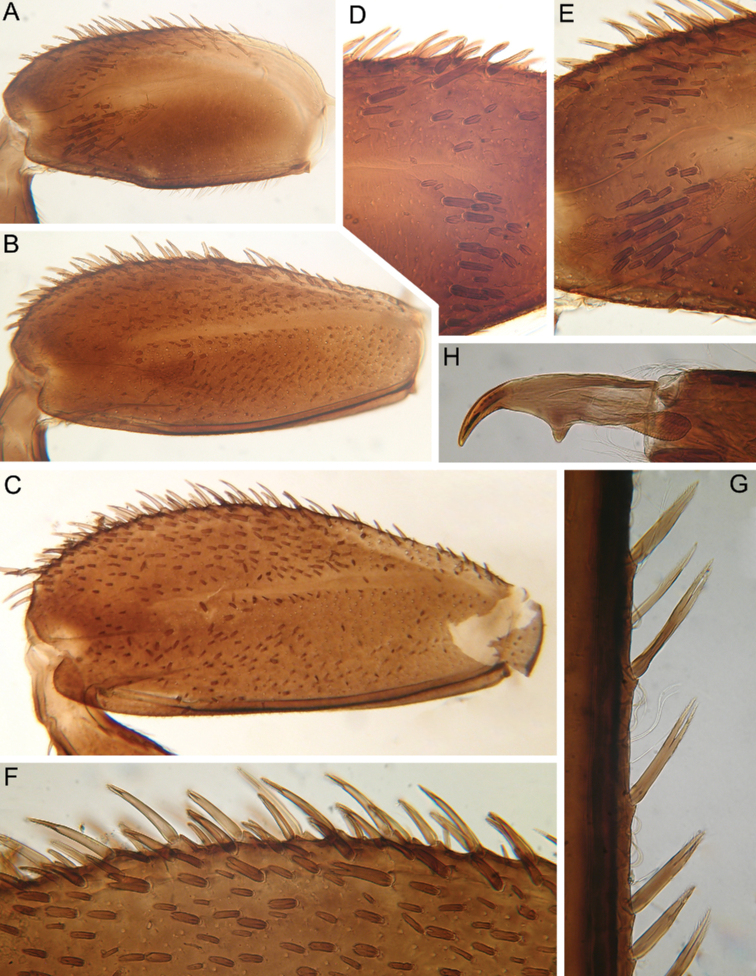
Larva of *Cincticostella shinichii* Martynov & Palatov, sp. nov., paratype (**A–C, E, F, H**) and holotype (**D, G**) **A** fore femur **B** middle femur **C** hind femur **D, E** stout setae of dorsal surface of fore femur **F** stout setae of outer margin of hind femur **G** stout setae of outer margin of middle tibia **H** tarsal claw.

**Figure 19.**
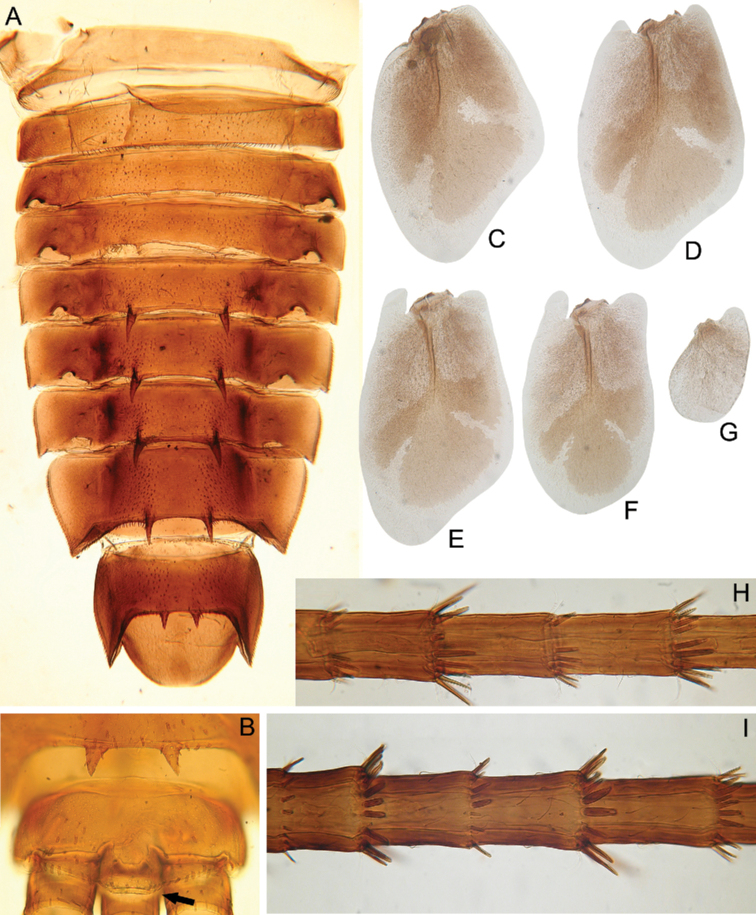
Larva of *Cincticostella shinichii* Martynov & Palatov, sp. nov., holotype (**A** and **I**) and paratype (**B–H**) **A** abdomen, dorsal view **B** tergum X and posterior margin of tergum IX (black arrow shows posterior margin of tergum X lack of paired submedian projections) **C–G** gills III–VII **H** caudal filament, middle part **I** caudal filament, proximal part.

Figs 16 , 17 , 18 , 19

#### Description.

**Larva.** Middle and late instars: body length of mature larva 12.0 mm, caudal filaments length 7.8 mm. Body yellowish-brown to brown, robust, covered with scale sockets and small scales in some of them.

***Head*:** Without tubercles and ridges. Genae moderately developed, rounded (Fig. 16A). Dorsal surface of head moderately covered with very short, elongated, rounded apically stout setae and few short hair-like setae. *Mouthparts* (Fig. 17A–G): Labrum wide, angles rounded; anteromedian emargination relatively deep and wide (labrum height in emargination/maximum labrum height ratio – 0.67–0.71) (Fig. 17C); anterior margin covered mainly with different-sized hair-like setae and several short, feathered setae in emargination. Surface of labrum densely covered with long hair-like setae, very short rounded scales with feathery margin and empty scale sockets. Mandibles covered with empty scale sockets, very short rounded scales with feathery margin and long hair-like setae, the greatest number of the hair-like setae on and near outer margin (Fig. 17A, B). Prostheca consisting of protuberance with tuft of middle-sized hair-like setae. Planate mandible with row of long thick hair-like setae (7–8) under the mola and tuft of middle-sized or short hair-like setae above; outer incisor with three teeth, inner incisor bifurcated. Angulate mandible with few short hair-like setae at the end of mola; outer incisor apex with four teeth; inner incisor with two distinct central teeth and one–two small lateral teeth. Hypopharynx with denser and longer setae on superlinguae apical part, lingua with short, hair-like setae (Fig. 17D); also, two subtransverse, submedian rows of short, pointed, stout setae (7–9) situated on lingua surface near base. Superlinguae with rounded apices. Maxilla (Fig. 17F) with two dentisetae with inner margins serrate. Apex and apical part of maxilla surfaces with numerous long, stout, hair-like setae; galea-lacinia with numerous long, stout and thin, hair-like setae on inner margin; also, in mature larvae galea-lacinia with group of 22–24 different-sized, stout, hair-like setae on surface near base. Maxillary palp long, 3-segmented (Fig. 17E). Segments I and II with long hair-like setae; most strong setae situated on segment I; segment III elongated and bluntly pointed apically, with several short fine setae. Joints of maxillary palp segments distinct. Labium (Fig. 17G) with rounded glossae; dorsal surface of glossae and apices of paraglossae covered mainly with long, stout, hair-like setae; whole ventral surface of labium covered mainly with long, stout, hair-like setae. Labial palp 3-segmented; segment I and segment II flattened and subequal in length; their ventral surfaces, outer and inner margins densely covered with long, stout, hair-like setae; central areas of dorsal surfaces of the segments almost without setae, only near margins long, stout, hair-like setae present; central part of dorsal surface of segment I with scale sockets and very short rounded scales with feathery margin in some of them. Segment III long (length/width ratio = 3.27–3.85, average – 3.53), rounded apically, covered with numerous short fine setae.

***Thorax*:** Dorsal surface of thorax covered with numerous small stout setae with divergent margins, rounded or bifurcated apices and less numerous short, strait, thin and stout, hair-like setae and scale sockets (Fig. 23A). Pronotum expanded laterally, with small, broad and rounded apically anterolateral projections (Fig. 16C–E). Anterolateral projections of mesothorax well-developed, with rounded posterior angles; outer margins not subparallel to lateral aspect of body, not notched, with very shallow sag only (Fig. 16B, C). Thoracic surface with small, indistinct ridges and tubercles. In mature larvae, paired posterior projections between fore wing pads small, rounded, but with deep and narrow cleft between them; apical parts of outer margins of projections not pressed against wing pads (Fig. 16C).

In late instars, femora of all legs slightly flattened (length/width ratio = fore femur 2.00–2.17; middle femur 2.17–2.33; hind femur 2.33–2.44) and bearing longitudinal ridge, especially visible on middle and hind femora (Fig. 18A–C). Average length ratio of leg’s parts (femur : tibia : tarsus): foreleg 1.95 : 1.68 : 1.00; middle leg 2.40 : 2.15 : 1.00; hind leg 2.70 : 2.84 : 1.00. Outer margins of all femora without apical projections.

Dorsal surface of fore femur with several elongated, pointed and bifurcated, stout setae with feathered margins near basal margin; also apical half of dorsal surface of fore femur with transverse, relatively wide and dense band of mainly middle-sized and short, bifurcated, bluntly pointed or rounded apically, stout setae (some situated on chalazae) (Fig. 18A, D, E). Basal half of inner margin of fore femur and adjacent part of dorsal surface with irregular row of spine-like setae and stout, hair-like setae. Outer margin of fore femur with numerous, mainly middle-sized and long, bifurcated apically, stout setae. Fore tibia and tarsus: outer margins of these with few hair-like setae (solitary and in tufts); inner margins with irregular, dense row of stout, hair-like setae. Dorsal surfaces of fore tibia with scattered hair-like setae and irregular, longitudinal row of long, stout setae with pointed and bifurcated apices; dorsal surface of fore tarsus with hair-like setae (solitary and in tufts) and long, stout hair-like setae.

Dorsal surface of middle and hind femora covered with numerous mainly middle-sized and short, rounded or bifurcated apically, stout setae (Fig. 18B, C). Except for the setae mentioned above, dorsal surface of all femora covered with scattered thin, hair-like setae and scale sockets with small scales in some of them. Inner margins of middle and hind femora with solitary hair-like setae only. Outer margins of middle and hind femora with irregular rows of mainly long, pointed and bifurcated, stout setae and few hair-like setae (Fig. 18B, C, F).

Outer margins of middle and hind tibiae with regular rows of long, pointed and bifurcated, stout setae and few hair-like setae (solitary and in tufts) amongst them (Fig. 18G). Dorsal surfaces of middle and hind tibiae and tarsi with hair-like setae (solitary and in tufts) and waved and hooked, stout, hair-like setae mainly. Dorsal surface of middle tibia also with row of long, pointed and bifurcated, stout setae near inner margin. Inner margin of middle and hind tibiae densely covered with long, stout hair-like setae and long, pointed or bifurcated, stout setae amongst them. Middle and hind tarsi: outer margins with hair-like setae (solitary and in tufts) only; inner margins densely covered with long, stout hair-like setae. Ventral surfaces of all tibiae and tarsi with numerous hair-like setae (solitary and in tufts) and long, thin, pointed, stout setae with feathered margins; stout setae situated in apical parts of tibiae and tarsi and along their inner margins.

Tarsal claws of all legs hooked, with one large denticle and several subapical setae (Fig. 18H).

***Abdomen*:** Central part of dorsal surface of terga II–X with two medial fields of middle-sized and small, stout setae with bifurcated, bluntly pointed or rounded apices (Fig. 19A). Surfaces of all terga also covered with scattered short, waved, stout hair-like setae, short, thin, hair-like setae, scale sockets and very short scales with feathered margins in some of them (greatest number of two last situated on lateral areas of tergal surfaces).

Posterior margins of tergum I with row of long, hair-like setae; of terga II–III (especially tergum II) with row of long, thin, bluntly pointed or rounded apically, stout setae and hair-like setae; of terga IV–VII with several elongated rounded or bifurcated apically stout setae near paired projections; of terga VIII–X with rows of elongated or short, stout setae with rounded or bifurcated apices (these rows extending from paired projections to lateral margins). Lateral margins of terga IV–VIII covered with numerous rounded or bifurcated apically stout setae. Posterolateral projections of abdominal segments IV–IX present (Fig. 19A).

Pairs of pointed, not bifurcated projections present on abdominal terga II–IX (Fig. 19A, B); projections on terga II–IV smaller than others; those on terga V–VIII strongest; projections on tergum IX elongated and distinctly pointed; posterior margin of tergum X smooth, without any projections. Surfaces of paired projections on terga V–IX covered with short stout setae with mainly rounded apices.

Dorsal surfaces of abdominal gills covered with hair-like setae (mainly in apical part) and scale sockets; shape of gills as in Fig. 19C–G; gill III without medial, transverse band of weakened membrane (Fig. 19C).

Caudal filaments subequal in length, with elongated stout setae with bluntly pointed, bifurcated or pointed apices and hair-like setae at articulations (Fig. 19H, I).

#### Adults.

Unknown.

#### Etymology.

The new species is named in honour of Dr. Shin-ichi Ishiwata (Kanagawa Environmental Research Center, Japan), who contributed significantly to the study of *Cincticostella* species.

#### Diagnosis.

This new species is close to *C. corpulenta*, but can be differentiated from this and other species of the complex by the following combination of characters: (i) genae moderately developed, rounded (Fig. 16A); (ii) labrum with deep anteromedian emargination (Fig. 17C); (iii) maxillary palp well-developed (Fig. 17E, F); (iv) segment III of maxillary palp small, bluntly pointed apically (Fig. 17E); (v) prothoracic anterolateral projections broad and rounded (Fig. 16C–E); (vi) mesothoracic anterolateral projections well-developed, with rounded posterior angles; outer margins not subparallel to lateral aspect of body, not notched, with very shallow sag only (Fig. 16B, C); (vii) small, stout setae with divergent margins, rounded or bifurcated apices present on dorsal surface of thorax (Fig. 23A); (viii) dorsal surface of fore femur with transverse, relatively wide and dense band of mainly middle-sized and short, bifurcated, bluntly pointed or rounded apically, stout setae (Fig. 18A, D, E); (ix) outer margins middle and hind femora with dense irregular rows of mainly middle-sized and long, stout setae (Fig. 18 B, C, F); (x) tarsal claw with one large denticle (Fig. 18H); (xi) pairs of pointed projections present on abdominal terga II–IX; projections on tergum IX elongated and distinctly pointed; tergum X without paired projections (Fig. 19A, B).

#### Distribution.

Known only from Nepal.

#### Habitat.

Larvae of *C. shinichii* sp. nov. were collected from stones with algal fouling in sections with current velocity of about 0.3–0.8 m/s of a large river (10–17 m wide) within low mountains on the southern slope of the Great Himalayan Range (Bagmati Zone, Central Nepal). Investigated rivers are under the significant anthropogenic pressure and can be classified as alpha- or beta-mezosaprobic waterbodies. Apparently, larvae inhabit rhithral zones of waterbodies with relatively high average current velocity and the predominance of large stones at the bottom. Larvae of *Stenopsyche*, Glossosomatidae, Hydropsychidae, *Baetis* (s.str.) sp. and representatives of other Ephemerellidae genera were collected along with the new species.

#### Type material.

***Holotype*: Nepal**: larva (on slide 647), Bagmati Zone, Shivapuri Nagarjun National Park, Melamchi River (1 km below Talamarang village), 27.844497°N, 85.557433°E, h ~ 900 m a.s.l., 4.iii.2007, Chertoprud M.V. leg. – *IN Nepa7Cinsp* [NMNHNASU]. ***Paratype*: Nepal**: larva (on slide 649), same data as holotype. – *IN Nepa7Cinsp* [NMNHNASU].

### Cincticostella wangi

Taxon classificationAnimaliaEphemeropteraEphemerellidae

Selvakumar, Martynov & Subramanian sp. nov.

7DD66255-1F03-5510-B16F-F9FE8C8C2F65

http://zoobank.org/C2DA1FE7-465B-4F1E-8F99-891714EF1B36

Figs 20 , 21

#### Description.

**Figure 20.**
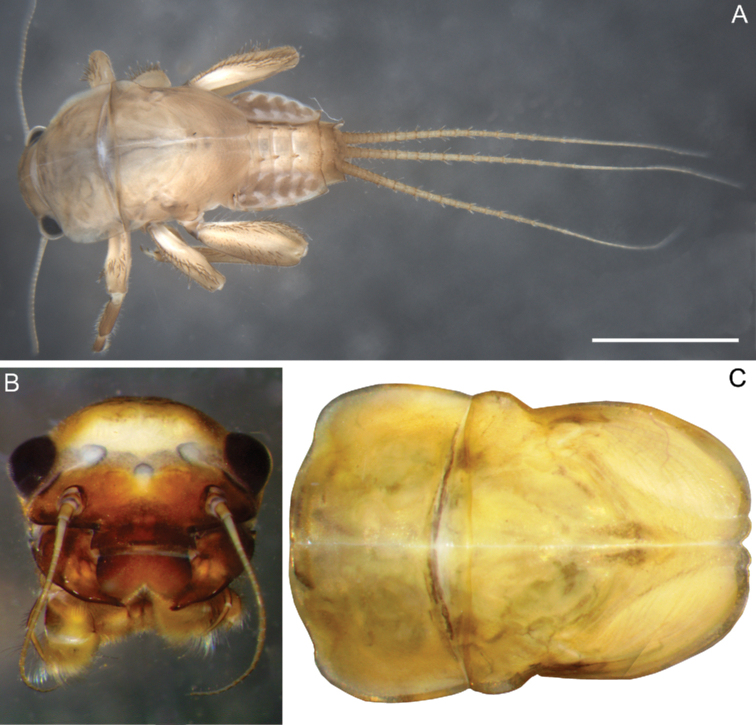
Larva of *Cincticostella wangi* Selvakumar, Martynov & Subramanian, sp. nov., holotype (**A**) and paratypes **(B** and **C) A** habitus, dorsal view, scale bar: 2 mm **B** head **C** thorax.

**Larva.** Body length 6.0–7.7 mm. Caudal filaments length 5.0–5.5 mm. Body colour yellowish-brown (Fig. 20A).

**Figure 21.**
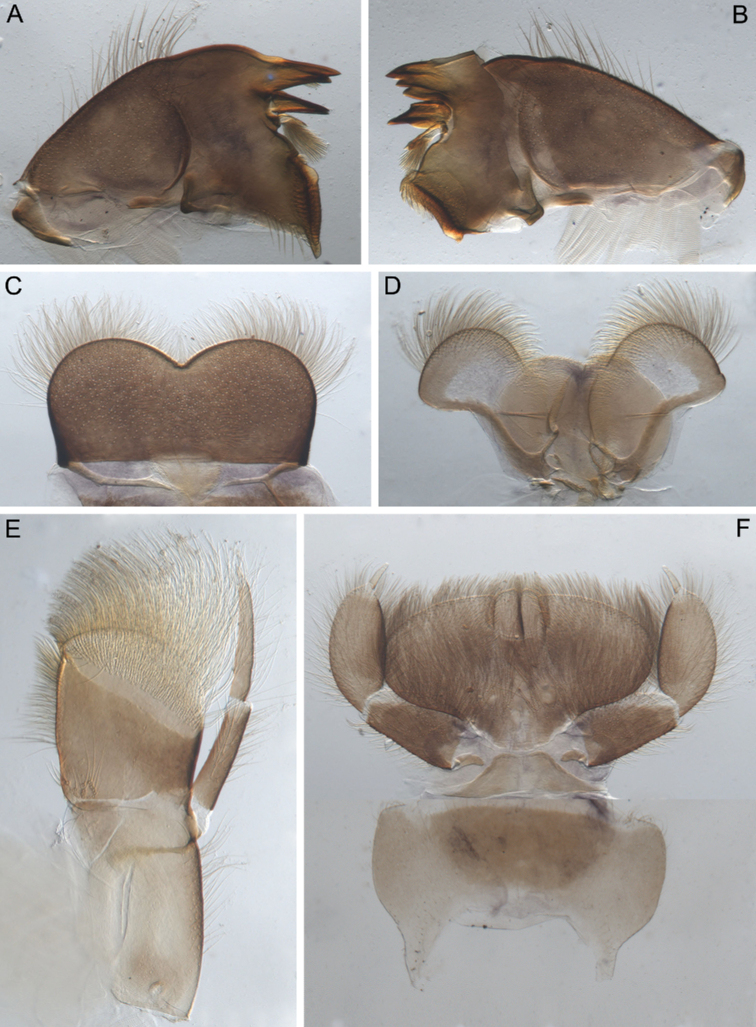
Larva of *Cincticostella wangi* Selvakumar, Martynov & Subramanian, sp. nov., paratypes **A** planate mandible **B** angulate mandible **C** labrum **D** hypopharynx **E** apical half of maxilla **F** labium.

***Head*:** Ocelli, clypeus dorsal surface and vertex without tubercles and ridges (Fig. 20B). Genae moderately developed. Anteromedian emargination of labrum deep and wide (labrum height in emargination/maximum labrum height ratio – 0.79) (Fig. 21C), anterior margin covered with long, thin and stout hair-like setae decreasing in length towards central notch. Dorsal surface of labrum densely covered with different hair-like setae, very short rounded scales and empty scale sockets.

Mandibles (Fig. 21A, B) with numerous long, stout hair-like setae in central part on dorsal surfaces and outer margins. Basal half of mandibles surfaces also covered with very short scales and empty scale sockets. Planate mandible: outer incisor with three teeth, inner incisor bifurcated; row of setae under mola consists of 8–9 long, stout hair-like setae. Angulate mandible with outer incisor consisting of four teeth, second one longer than others, inner incisor with three–four teeth, central ones distinctly larger. Superlinguae of hypopharynx with numerous long, stout hair-like setae on apices; lingua with very sparse and tiny setae on dorsal and ventral surfaces, anterior margin with shallow medial concave (Fig. 21D). Lingua surface near base bears irregular rows of short, pointed, stout setae (7–9); these rows subtransverse relative to the longitudinal axis of body. Maxilla (Fig. 21E) with two dentisetae; apex of maxilla with bushy group of long stout and thin hair-like setae; inner margin of galea-lacinia with dense row of long, stout hair-like setae; surface galea-lacinia base with few, mainly long and middle-sized, stout hair-like setae near inner margin. Maxillary palp well-developed, 3-segmented, with segments I and II subequal in length; segment I slightly broader than segment II, segment III short and pointed. Joints between maxillary palp segments distinct (Fig. 21E). Segments I and II with numerous long, stout hair-like setae. Labium (Fig. 21F): ventral surface densely covered with long, stout and thin hair-like setae; dorsal surface of glossae and apices of paraglossae covered by similar setae. Labial palp 3-segmented; segments I and II flattened and subequal in length; covered with long, stout and thin, hair-like setae on ventral surfaces; segment I with group of scale sockets and scales in some of them in central part of dorsal surface. Segment III rounded apically, elongated (length/width ratio = 3.0–3.5); apex covered with few short, fine setae.

***Thorax*:** Pronotum expended laterally, with anterolateral angles small and projecting forward (Fig. 20C); mesonotum projections moderately developed, rounded, their outer margins not notched. Dorsal surface of thorax with scattered short, relatively strait, stout hair-like setae (Fig. 23C); thoracic nota without ridges or projections. Paired posterior projections between fore wing pads, moderately developed, rounded, cleft between them shallow and smooth; apical parts of outer margins of projections not pressed against wing pads.

**Figure 22.**
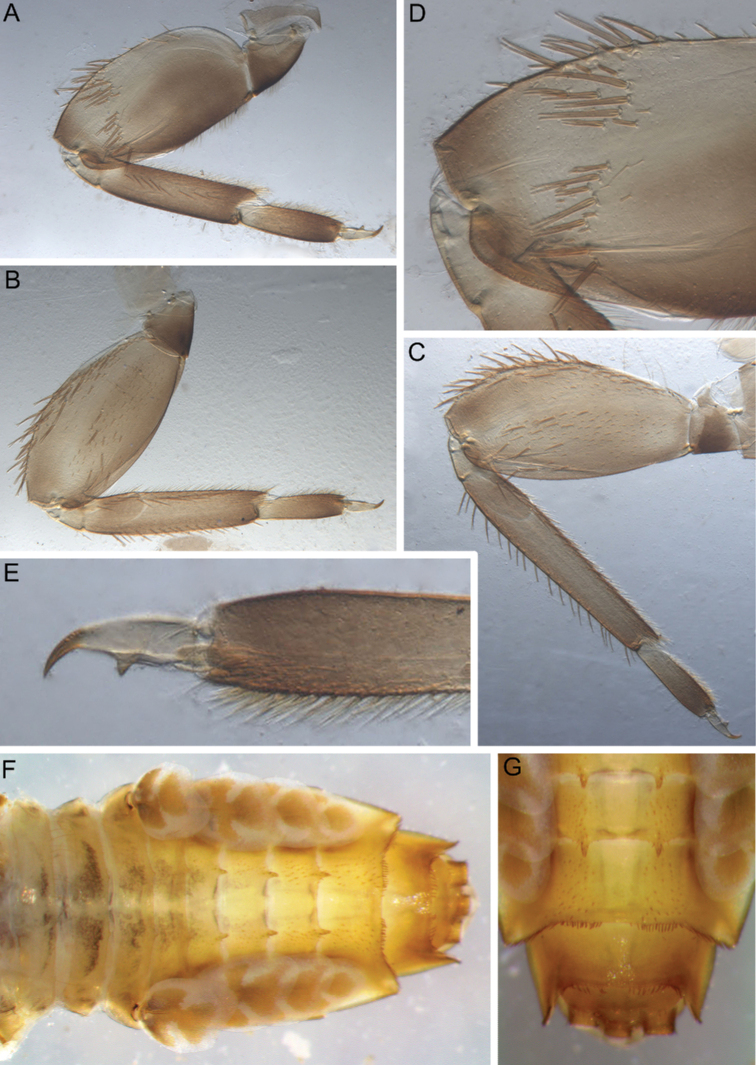
Larva of *Cincticostella wangi* Selvakumar, Martynov & Subramanian, sp. nov., paratypes **A** fore femur **B** middle femur **C** hind femur **D** stout setae of dorsal surface of fore femur **E** tarsal claw and tarsus **F** abdomen, dorsal view **G** terga VI–X.

Femora of all legs flattened (length/width ratio = fore femur 2.0–2.1; middle femur 2.0–2.1; hind femur 2.0–2.2), with longitudinal ridges (Fig. 22A–C). Femora of all legs longer than tibiae and tibiae longer than tarsi. Outer margins of all femora without apical projections.

Fore femur with distinct transverse band of numerous, mainly extremely long to middle-sized, stout setae with deeply bifurcated apices (some situated on chalazae) (Fig. 22A, D); another part of dorsal surface with scattered hair-like setae and several pointed, stout setae in basal area and along outer margin. Outer margin of fore femur with few stout, bifurcated and pointed, stout setae; main number of the setae situated in transverse row area. Basal half of inner margin and adjacent area of dorsal surface densely covered with spine-like setae and stout hair-like setae.

Dorsal surfaces of middle and hind femora almost completely covered with different-sized, bifurcated, stout setae, some very long (Fig. 22B, C); only longitudinal ridges and distal part of surfaces from ridges to outer margins without these stout setae. In addition, scattered hair-like setae, very small scales and empty scale sockets cover all dorsal surfaces of middle and hind femora. Outer margins of middle and hind femora covered with long, bifurcated stout setae decreasing in length towards base of femora; basal half of outer margins also with long, stout hair-like setae. Inner margins of middle and hind femora without stout setae, only with scattered hair-like setae.

Dorsal surface of all tibiae with longitudinal rows of long, bifurcated stout setae; in middle and hind legs, these rows situated closer to inner margins of tibiae. Outer margin of fore tibia with thin hair-like setae only; in middle and hind tibiae also with regular rows of long, bifurcated, stout setae.

Claws of all legs with one denticle each and several subapical setae (Fig. 22E).

***Abdomen*:** Paired, pointed projections present on abdominal terga II–VIII; posterior margin of tergum VIII almost straight, its protuberances very small and smooth; posterior margin of terga IX and X without projections (Fig. 22F, G). Dorsal surfaces of terga above projections and surfaces of projections covered with short stout setae. Posterior margins of terga VIII–IX in submedian areas covered with rows of elongated, rounded apically, stout setae; posterior margin of tergum X with a discontinuous row of stout setae.

Gill III without medial transverse band of weakened membrane, dorsal lobe of gills III–V similar in shape (Fig. 22F), gill VI diminished in size compared to gills III–V, gill VII very small and wholly covered by gill VI.

Caudal filaments subequal in length, with pointed, stout setae and hair-like setae on posterior edge of each segment, setae shorter than corresponding segment.

**Figure 23.**
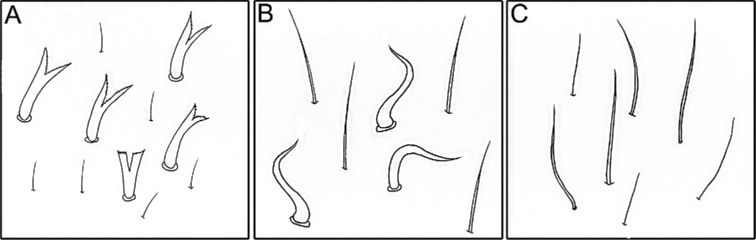
Setae on dorsal surface of thorax of some representatives of *Cincticostella nigra* (Uéno, 1928) complex **A** dominant kind of stout setae – numerous small stout setae with divergent margins, bifurcated apices **B** dominant kind of stout setae – short, waved and hooked, stout hair-like setae **C** dominant kind of stout setae – scattered short, relatively strait, stout hair-like setae.

#### Adults.

Unknown.

#### Etymology.

The new species is named in honour of Dr. T.-Q. Wang (formerly Purdue University, USA), who contributed significantly to the study of Ephemerelloidea.

**Figure 24.**
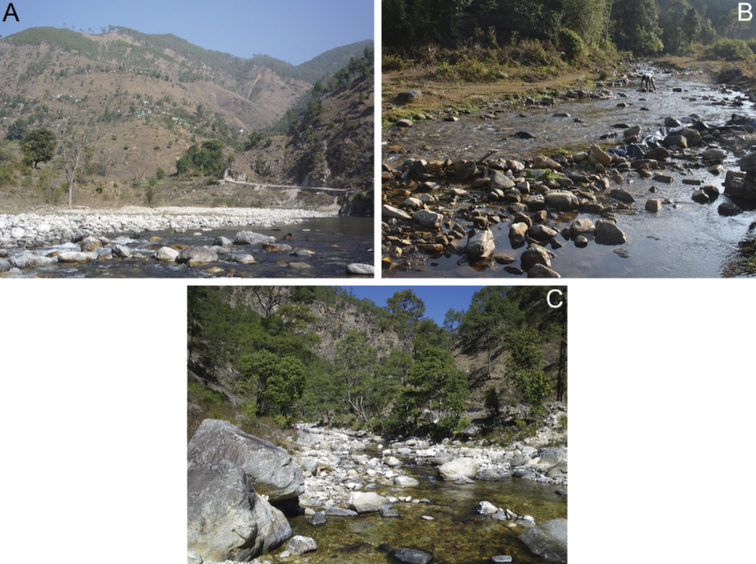
Habitats of *Cincticostella changfai* Martynov & Palatov, sp. nov. (**A**) and *Cincticostella funki* Martynov, Selvakumar, Palatov & Vasanth sp. nov. (**B** and **C**). **A** Ramganga River (300 m above Patangoari Village), Uttarakhand State, Almora District, India **B** Rike River (vicinity of Parang Village), Arunachal Pradesh, Papumpare District, India **C** second order left tributary of the Ramganga River (10.1 km north-eastwards of Chaukhutia Town), Uttarakhand State, Almora District, India.

#### Diagnosis.

This species can be distinguished from other representatives of *C. nigra* complex by the following combination of characters: (i) genae moderately developed, rounded; (ii) labrum with deep and wide anteromedian emargination (Fig. 21C); (iii) maxillary palp well-developed (Fig. 21E); (iv) segment III of maxillary palp small, pointed; (v) prothoracic anterolateral projections small projecting forward (Fig. 20C); (vi) mesonotum projections mesonotum projections moderately developed, rounded, their outer margins not notched (Fig. 20C); (vii) dorsal surface of thorax with scattered short, relatively strait, stout hair-like setae (Fig. 23C); (viii) fore femur with distinct transverse band of numerous, mainly extremely long to middle-sized, stout setae with deeply bifurcated apices (Fig. 22A, D); (ix) tarsal claw with one denticle (Fig. 22E); (x) paired, pointed projections present on abdominal terga II–VIII; posterior margin of tergum VIII with very small and smooth protuberances; posterior margin of terga IX and X without projections (Fig. 22F, G); (xi) posterior margins of terga VIII–IX in submedian areas covered with rows of elongated, rounded, stout setae (Fig. 22F, G).

#### Distribution.

India-China border region.

#### Habitat.

Cold fast-flowing stream with cobbles and gravel. The type locality of *C. wangi* sp. nov. is shown in Martynov et al. (2019: fig. 151).

#### Type material.

***Holotype*: India**: larva, Arunachal Pradesh, Lower Subansiri District, Tale Valley, unnamed stream, 27.537201°N, 93.959883°E, h ~ 2370 m a.s.l., 14.iv.2015, Coll. K.A. Subramanian – *IN 5575/H13* [ZSI]. ***Paratypes*: India**: 8 larvae, same data as holotype – *IN 5575/H13* [ZSI].

### Cincticostella

Taxon classificationAnimaliaEphemeropteraEphemerellidae

sp. A

848326E2-530B-5598-90E4-3FC63E4AE4D7

#### Diagnosis.

Immature larva: (i) genae moderately developed, rounded; (ii) anteromedian emargination of labrum shallow (labrum height in emargination/maximum labrum height ratio – 0.85); (iii) maxillary palp well-developed; (iv) segment III of maxillary palp, thin, elongated, rounded apically; (v) group of 13–16 setae different-sized, stout, hair-like situated on surface of galea-lacinia near base; (vi) prothoracic anterolateral projections small; (vii) mesothoracic anterolateral projections well-developed, subparallel to lateral aspect of body, not notched; (viii) surface of thorax covered with scattered short, thin, hair-like setae and few scale sockets and very short rounded scales with feathered margins in some of them; few waved and hooked setae cover mainly wing pads; (ix) dorsal surface of fore femur with sparse, transverse row of mainly long, stout setae with slightly convergent margins and bifurcated apices; (x) tarsal claw with one large denticle and several subapical setae; (xi) pairs of pointed projections present on abdominal terga II–IX; those on terga V–VIII strongest; those on tergum IX moderately developed, rounded apically.

#### Distribution.

Nepal.

#### Remarks.

Two middle larval instars (body length 5.5–6.5 mm; caudal filaments length 5.2–5.3 mm) of this operational taxonomic unit (OTU) were collected in Nepal in 2014. However, the poor material and absence of late larval instars do not allow us to describe a new species adequately. We hope that this account will facilitate discovery of additional material of this OTU.

#### Habitat.

Larvae of *Cincticostella* sp. A inhabit cold, moderate flowing springs with stones, gravel and clumps of moss. Larvae were collected from such a spring (up 1.3 m wide) in medium high mountains, on the southern slope of the Great Himalayan Range. This was a small, lotic waterbody with relatively low water temperature (10 °C in sampling period), average current velocity (0.2–0.6 m/s), mosaic bottom and a low degree of anthropogenic pressure.

#### Material examined.

**Nepal**: 2 larvae (one on slide 634), Gandaki Zone, Kaski District, stream at the Tolka-Lambruk Road, 28.365000°N, 83.831667°E, h ~ 2000 m a.s.l., 27.i.2014, Chertoprud M.V., Marinskiy V.V. leg. – *IN Nepa1Cinsp* [NMNHNASU].

##### *Cincticostella* of India and southern limit of the genus distribution

To date, nine species of the genus *Cincticostella* are known from India: *C. bifurcata* Xie, Jia, Chen, Jacobus & Zhou, 2009; *C. braaschi* Jacobus & McCafferty, 2008; *C. changfai* Martynov & Palatov, sp. nov.; *C. funki* Martynov, Selvakumar, Palatov & Vasanth sp. nov.; *C. gosei* (Allen, 1975); *C. insolta* (Allen, 1971); *C. ranga* Selvakumar & Subramanian, 2019; *C. richardi* Martynov & Palatov, 2019 and *C. wangi* Selvakumar, Martynov & Subramanian, sp. nov. (Martynov et al. 2019, new data). Distribution of the species within India falls within the Himalayan Region. In all known cases, the species inhabit coldwater streams and rivers with significant current velocity and coarse substrate.

Overall, only three of all known species of *Cincticostella* occur at latitudes that are more southern than the Himalayan Region, namely *C. gosei*, *C. insolta* and *C. femorata*. The southernmost records of *C. insolta* are from Thailand, where the species inhabits coldwater flows in northern upland region of the country (Martynov et al. 2019). *Cincticostella femorata* inhabits similar waterbodies in the Region (Martynov et al. 2019; Zheng and Zhou 2021). However, this species and *C. gosei* penetrate southwards in Thailand, where they would seem to prefer warmer biotopes (e.g. Gose 1969). These records form the southern border of the genus distribution at about 12.6°N latitude.

##### Species complexes in *Cincticostella*

*Cincticostella* comprises at least 21 valid species (Table 2) (Jacobus and McCafferty 2008; Xie et al. 2009; Martynov et al. 2019; Auychinda et al. 2020; new data), but it remains poorly investigated. For the vast majority of species, only the larval stage is known – as is the case for all representatives of the *C. insolta* complex, except for *C. femorata* (Zhang et al. 2020; Zheng and Zhou 2021).

Allen (1975) divided all representatives of the genus into two species groups, based on larval characters: the *nigra*-group and the *insolta*-group. According to Allen (1975), representatives of the *nigra*-group lack head tubercles and their middle and hind pairs of femora are narrow, not enlarged and the margins are without serration; the *insolta*-group was characterised by possessing suboccipital head tubercles and the middle and hind pairs of femora being enlarged with serrated margins and/or protuberances. Later, the *insolta*-group was designated as the separate subgenus Rhionella Allen, 1980; all other species, including *C. gosei*, were placed in *Cincticostella* s.str. (Allen 1980). In the revision of Ephemerellidae genera, *Rhionella* was strictly synonymised with *Cincticostella* (Jacobus and McCafferty 2008), based on the position of its type species within the tree of other *Cincticostella* species (fig. 94 in Jacobus and McCafferty 2008).

In light of the several new species described since Jacobus and McCafferty (2008) revised the generic classification of the group and considering possible polyphyly, we re-evaluated the larval morphology of *Cincticostella* species for understanding the difference of complexes and expediency of their designation; available material and literature data were used for this analysis (Tshernova 1952; Allen 1975; Braasch 1981; Kang and Yang 1995; Ishiwata 2003; Jacobus and McCafferty 2008; Xie et al. 2009; Martynov et al. 2019; Auychinda et al. 2020; Zhang et al. 2020; Zheng and Zhou 2021).

Based on our research, we conclude that the establishment of the discussed complexes was for expediency. In fact, the *C. insolta* and *C. nigra* complexes are more distanced morphologically from each other than previously thought (see Allen 1975), based on our new distinguishing characters (see characters 3–10 in Table 1, below). All but one species of *Cincticostella* clearly fit into the *C. insolta* or *C. nigra* complexes as here defined; only *C. gosei* has a combination of characters that does not completely fit into any of these two complexes. Therefore, the monotypic *C. gosei* complex is proposed herein (Tables 1 and 2). We acknowledge possible paraphyly of the other complexes.

**Table 1.** Distinguishing larval characters of *Cincticostella* species complexes.

	Characters	*C. insolta* complex	*C. nigra* complex	*C. gosei* complex
1	Two pairs of suboccipital tubercles	present	absent	absent
2	Serration of margins of middle and hind femora	present	absent	absent*
3	Presence of numerous large, rounded, scale sockets on body surface	present	absent	present
4	Rate of anterolateral emargination of labrum	shallow	from shallow to deep	moderate
5	Maxillary palp	reduced, articulations of segments not distinct, especially between segments I and II	mainly well-developed, articulations of all segments distinct**	absent
6	Segments I and II of labial palp	wide	wide	relatively narrow, elongated
7	Stout setae on outer margin of fore femur	several stout setae only	numerous stout setae	several stout setae only
8	Stout setae on dorsal surface of middle and hind femora	absent or up to several stout setae in basal area	surface with numerous stout setae	absent
9	Shape of hind femur	strongly or moderately widened	moderately widened	moderately widened
10	Stout setae on dorsal surface of abdominal terga and paired projections	absent	present	absent

**Notes**: *Gose’s (1969: figs 50 and 51) text and illustrations, which became the basis for the *C. gosei* description (Allen 1975), indicate that the outer margins of middle and hind femora have distinct serration; however, all specimens investigated by us (Fig. 15B, C) lacked distinct serrations on the femora. **According to Ishiwata (2003), specimens of *C. orientalis* from Japan have the maxillary palp reduced, but with distinct articulations of segments; specimens studied by us from Russia (Primorsky Krai) had the maxillary palp reduced, without distinct articulations.

The following morphological features place *C. gosei* closer to the *C. insolta* complex than to the *C. nigra* complex: presence of numerous large, rounded, scale sockets on body surface, absence of maxillary palp (this character state may prove to be related to significant reduction of the maxillary palp, including reduction of segments’ articulations), number of stout setae on the outer margin of the fore femur and absence of numerous stout setae on dorsal surfaces of the middle and hind femora, complete absence of stout setae on dorsal surfaces of abdominal terga and paired projections.

**Table 2.** Systematic structure of *Cincticostella*.

*C. insolta* complex	*C. gosei* complex	*C. nigra* complex
*C. bifurcata* Xie, Jia, Chen, Jacobus & Zhou, 2009	*C. gosei* (Allen, 1975)	*C. changfai* Martynov & Palatov, sp. nov.
*C. braaschi* Jacobus & McCafferty, 2008		*C. colossa* Kang & Yang, 1995
*C. femorata* (Tshernova, 1972)		*C. corpulenta* (Braasch, 1981)
*C. insolta* (Allen, 1971)		*C. elongatula* (McLachlan, 1875)
*C. ranga* Selvakumar & Subramanian, 2019		*C. funki* Martynov, Selvakumar, Palatov & Vasanth sp. nov.
*C. richardi* Martynov & Palatov, 2019		*C. fusca* Kang & Yang, 1995
*C. sivaramakrishnani* Martynov & Palatov, 2019		*C. levanidovae* (Tshernova, 1952)
*C. tornata* Auychinda & Gattolliat, 2020		*C. nigra* (Uéno, 1928)
		*C. orientalis* (Tshernova, 1952)
		*C. shinichii* Martynov & Palatov, sp. nov.
		*C. szechuanensis* Xie, Jia, Chen, Jacobus & Zhou, 2009
		*C. wangi* Selvakumar, Martynov & Subramanian, sp. nov.
		*Cincticostella* sp. A

Ogden et al. (2009) included only two species from this genus in their combined evidence for phylogenetic analyses of Ephemerellidae. Further investigation of imaginal characters after new stage associations (e.g. Zhang et al. 2020; Zheng and Zhou 2021) and molecular investigation (Martynov, in progress) will help to better understand the structure and relationships of species in the genus *Cincticostella*.

## Supplementary Material

XML Treatment for Cincticostella changfai

XML Treatment for Cincticostella corpulenta

XML Treatment for Cincticostella funki

XML Treatment for Cincticostella gosei

XML Treatment for Cincticostella indica

XML Treatment for Cincticostella shinichii

XML Treatment for Cincticostella wangi

XML Treatment for Cincticostella
